# A Novel Late-Stage Autophagy Inhibitor That Efficiently Targets Lysosomes Inducing Potent Cytotoxic and Sensitizing Effects in Lung Cancer

**DOI:** 10.3390/cancers14143387

**Published:** 2022-07-12

**Authors:** Adrià Molero-Valenzuela, Pere Fontova, Daniel Alonso-Carrillo, Israel Carreira-Barral, Ana Aurora Torres, María García-Valverde, Cristina Benítez-García, Ricardo Pérez-Tomás, Roberto Quesada, Vanessa Soto-Cerrato

**Affiliations:** 1 Department of Pathology and Experimental Therapeutics, Faculty of Medicine and Health Sciences, Universitat de Barcelona, L’Hospitalet de Llobregat, 08907 Barcelona, Spain; adria.molero@vhir.org (A.M.-V.); atorrega30@alumnes.ub.edu (A.A.T.); cbenitezg16@ub.edu (C.B.-G.); rperez@ub.edu (R.P.-T.); 2 Department of Chemistry, Universidad de Burgos, 09001 Burgos, Spain; pfontova@ubu.es (P.F.); dacarrillo@ubu.es (D.A.-C.); icarreira@ubu.es (I.C.-B.); magaval@ubu.es (M.G.-V.); rquesada@ubu.es (R.Q.); 3 Molecular Signalling, Oncobell Program, Institut d’Investigació Biomèdica de Bellvitge (IDIBELL), L’Hospitalet de Llobregat, 08908 Barcelona, Spain

**Keywords:** autophagy, treatment resistance, autophagy inhibitor, lysosomal dysfunction, anionophore, lung cancer, treatment sensitization

## Abstract

**Simple Summary:**

Lung cancer is the main cause of cancer-related deaths worldwide, mainly due to treatment resistance. For that reason, it is necessary to develop novel therapeutic strategies to overcome this phenomenon. The aim of our study was to design and characterize a synthetic anionophore, LAI-1, that would be able to efficiently disrupt lysosomal activity, leading to autophagy blockage, one of the most important resistance mechanisms in cancer cells. We confirmed that LAI-1 selectively localized in lysosomes, deacidifying them. This effect produced a blockage of autophagy, characterized by an abrogation of autophagosomes and lysosomes fusion. Moreover, LAI-1 produced cell death in lung cancer cells from different histological subtypes, inducing cytotoxicity more efficiently than other known autophagy inhibitors. Finally, LAI-1 was evaluated in combination therapy, showing sensitization to the first-line chemotherapeutic agent cisplatin. Altogether, LAI-1 is a novel late-stage autophagy inhibitor with potential therapeutic applications in tumors with cytoprotective autophagy.

**Abstract:**

Overcoming resistance is one of the most challenging features in current anticancer therapy. Autophagy is a cellular process that confers resistance in some advanced tumors, since it enables cancer cells to adapt to stressful situations, such as anticancer treatments. Hence, the inhibition of this cytoprotective autophagy leads to tumor cells sensitization and death. In this regard, we designed a novel potent anionophore compound that specifically targets lysosomes, called LAI-1 (late-stage autophagy inhibitor-1), and evaluated its role in blocking autophagy and its potential anticancer effects in three lung cancer cell lines from different histological subtypes. Compared to other autophagy inhibitors, such as chloroquine and 3-Methyladenine, the LAI-1 treatment induced more potent anticancer effects in all tested cancer cells. LAI-1 was able to efficiently target and deacidify lysosomes, while acidifying cytoplasmic pH. Consequently, LAI-1 efficiently blocked autophagy, indicated by the increased LC3-II/I ratio and p62/SQSTM1 levels. Moreover, no colocalization was observed between autophagosomes, marked with LC3 or p62/SQSTM1, and lysosomes, stained with LAMP-1, after the LAI-1 treatment, indicating the blockage of autophagolysosome formation. Furthermore, LAI-1 induced cell death by activating apoptosis (enhancing the cleavage of caspase-3 and PARP) or necrosis, depending on the cancer cell line. Finally, LAI-1 sensitized cancer cells to the first-line chemotherapeutic agent cisplatin. Altogether, LAI-1 is a new late-stage autophagy inhibitor that causes lysosomal dysfunction and the blockage of autophagolysosome formation, as well as potently induces cancer cell death and sensitization to conventional treatments at lower concentrations than other known autophagy inhibitors, appearing as a potential new therapeutic approach to overcome cancer resistance.

## 1. Introduction

Lung cancer is the second most frequent cancer worldwide, with over two million people diagnosed every year, and is the leading cause of cancer death, accounting for 18.4% of the total cancer-related deaths [1]. Poor prognosis in lung cancer is partly due to a late diagnosis that results from the lack of any obvious symptoms during early disease progression. Thus, most lung tumors are diagnosed in advanced stages, and improving survival is a major challenge for modern clinical oncology, considering that the 5-year survival rate remains lower than 15% across all stages of the disease [2]. According to the World Health Organization histological classification published in 2015, two main lung cancer types can be distinguished: small-cell lung carcinoma (SCLC) and non-small-cell lung carcinoma (NSCLC) [3]. NSCLC accounts for approximately 80–85% of all lung cancer cases, and it is further subdivided into three histological subtypes, including adenocarcinoma, squamous-cell carcinoma and large-cell carcinoma [4].

Although cancer therapy has significantly improved in recent decades, drug resistance remains one of the most important limitations to achieve cures in cancer patients, leading to recurrence and death [5,6]. This is partly due to cancer heterogeneity, common to many tumors, which enables tumor survival against treatments through the activation of different pathways, along with tumoral microenvironment plasticity, which allows tumor cells to adapt to stress situations [7].

One of the cellular mechanisms that can confer treatment resistance to tumor cells is called autophagy [8,9]. The autophagy process consists of a highly conserved catabolic mechanism that regulates cellular homeostasis in front of extracellular and intracellular stresses, such as growth factor deprivation, nutrient starvation and hypoxia or protein aggregation, through the degradation and recycling of cytoplasmic components in lysosomes [10,11]. The autophagy pathway has multiple steps, including initiation, nucleation, elongation, maturation and vesicle content degradation, which are tightly regulated by a family of proteins called autophagy-related proteins (ATGs) [9,12]. Moreover, other proteins, such as the microtubule-associated protein light chain 3 (LC3) and p62/Sequestosome-1 (SQSTM1), are crucial for the correct functioning of the process. LC3 is cleaved by a protease during the elongation phase to form LC3-I; then, it is activated by ATG7 and ATG3, promoting the assembly with membrane-resident phosphatidylethanolamine (PE) to form LC3-II, which allows the vesicle to elongate and fuse [13,14]. On the other side, p62/SQSTM1 is one of the cargo recognition regulators, as it delivers polyubiquitinated cargos to autophagosomes through its ATG8 family-interacting motif. At the end of the process, p62/SQSTM1 is degraded along with the cargo into the autophagolysosome [15,16].

Autophagy has a dual role in cancer development. On the one hand, in a healthy tissue context, it has the mentioned physiological homeostatic role, acting in response to different stresses and preventing tumor initiation [17]. On the other hand, once the malignant tumor is formed, autophagy activation allows some tumors to escape from anticancer therapies or other stresses by enhancing cell adaptation, promoting tumor progression and cancer recurrence [18]. Therefore, the inhibition of autophagy in this kind of tumor may be a promising therapeutic strategy to treat cancer or sensitize tumors to conventional treatments. In fact, the well-known autophagy inhibitor chloroquine (CQ) has already been used in combination with conventional chemotherapy, showing promising results [19].

In this view, we designed a novel potential late-stage autophagy inhibitor, called LAI-1, that specifically targets lysosomes and exerts an anionophoric activity across membranes, with the aim of inducing a blockage of autophagy, leading to cancer cell death and treatment sensitization. The evaluation of its molecular mechanism of action and its anticancer effects in several lung cancer cell lines with different histological and genomic backgrounds was evaluated in depth to elucidate which kind of tumor may be more sensitive to this therapeutic approach and reveal the potential of this compound as a new autophagy inhibitor with anticancer properties.

## 2. Materials and Methods

### 2.1. Reagents

Chloroquine (CQ) and 3-Methyladenine (3-MA) from Sigma-Aldrich (St Louis, MO, USA) were used. Late-stage autophagy inhibitor-1 (LAI-1) was synthesized as explained in the following section. CQ and LAI-1 were stocked at 10 mM in water and DMSO, respectively, and 3-MA was dissolved at 50 mM in RPMI and stocked at −20 °C and warmed to be redissolved before use.

### 2.2. LAI-1 Synthesis

LAI-1 was synthesized by acid-catalyzed condensation of 3-methoxy-5-(1-(4-(2-morpholinoethyl)phenyl)-1*H*-1,2,3-triazol-4-yl)-1*H*-pyrrole-2-carbaldehyde and 1-adamantylamine in chloroform. LAI-1 was obtained as a dark brown non-crystalline solid isolated as a dihydrochloride salt. Full experimental details and characterization data are provided as Supplementary Materials (Figures S1–S11).

### 2.3. Transmembrane Anion Transport Experiments in Vesicles

Anion transport activity of LAI-1 was evaluated in unilamellar 1-palmitoyl-2-oleoyl-*sn*-glycero-3-phosphocholine (POPC) vesicles (mean diameter: 200 nm). Vesicles containing a NaCl aqueous solution (489 mM NaCl, 5 mM NaH_2_PO_4_, I.S. 500 mM, pH 7.2, for Cl^−^/NO_3_^−^ exchange experiments, or 451 mM NaCl, 20 mM NaH_2_PO_4_, I.S. 500 mM, pH 7.2, for Cl^−^/HCO_3_^−^ exchange experiments) were dispersed in a NaNO_3_ aqueous solution (489 mM NaNO_3_, 5 mM NaH_2_PO_4_, I.S. 500 mM, pH 7.2, for Cl^−^/NO_3_^−^ exchange experiments) or a Na_2_SO_4_ aqueous solution (150 mM Na_2_SO_4_, 20 mM NaH_2_PO_4_, I.S. 500 mM, pH 7.2, for Cl^−^/HCO_3_^−^ exchange experiments), the final lipid concentration during the assays being 0.5 mM and the final volume being 5 mL. A certain volume of a solution of the LAI-1 compound in DMSO (or the blank DMSO, 12.5 μL) was added at t = 0 s, and the chloride released was monitored for 300 s with a chloride-selective electrode (HACH 9652C). At t = 300 s, a surfactant (Triton-X, 20% dispersion in water, 20 µL) was added to lyse the vesicles and release all the encapsulated chloride. This value was considered 100% chloride release and employed as such. Regarding the Cl^−^/HCO_3_^−^ exchange assays, a 500 mM NaHCO_3_ aqueous solution prepared with the Na_2_SO_4_ one (150 mM Na_2_SO_4_, 20 mM NaH_2_PO_4_, I.S. 500 mM, pH 7.2) was added at t = −10 s to the vesicle suspension, with the HCO_3_^−^ concentration during the assay being 40 mM. The rest of the experimental procedure was similar to that described above. Experimental details of HPTS- and carboxyfluorescein-based assays can be found in Supplementary Materials.

### 2.4. Cell Culture

Human lung cancer cell lines A549 (adenocarcinoma, KRAS-mutated), DMS53 (small cell carcinoma, p53-mutated) and SW900 (squamous carcinoma, KRAS- and p53-mutated) were obtained from the American Type Culture Collection (ATCC, Manassas, VA, USA), and maintained in DMEM (A549) or RPMI (DMS53 and SW900) medium (Biological Industries, Beit Haemek, Israel). All media were supplemented with 100 U/mL penicillin, 100 g/mL streptomycin and 2 mM L-glutamine, all from Biological Industries, and 10% of fetal bovine serum (FBS; Gibco, Paisley, UK). Cell lines were grown at 37 °C in a humidified incubator (Thermo Fisher Scientific Inc, Waltham, MA, USA) with 5% CO_2_ atmosphere. All cell lines were seeded and incubated for 24 h to attach them to their corresponding complete medium before the different treatment conditions used in the diverse techniques.

### 2.5. Cell Viability

Cell viability was determined with the MTT assay (3-(4,5-dimethylthiazol-2-yl)-2,5-diphenyltetrazolium bromide). Cells (1 × 10^5^ cells/mL) were seeded in 96-well microtiter plates and incubated for 24 h to allow cells to attach. Afterwards, cells were treated for 24 h with three different compounds, two well-known autophagy inhibitors CQ and 3-MA, and our new compound LAI-1 for dose–response curves using water, medium and DMSO as negative controls, respectively. For combination therapy experiments, all cell lines were treated with 40 µM of cisplatin (CisPt), a chemotherapeutic agent, and 15 µM of LAI-1. After 24 h of treatment, 10 μM of MTT (Sigma-Aldrich) diluted in 1X PBS was added to each well for an additional 2 h. The medium was removed and the MTT formazan precipitate was dissolved in 100 μL of DMSO. Absorbance was read on a Multiskan multiwell plate reader (Thermo Fisher Scientific) at 570 nm. The inhibitory concentration (IC) values of 25% (IC_25_), 50% (IC_50_) and 75% (IC_75_) of the cell population were calculated with GraphPad Prism^TM^ v8 software (Graph Pad Software, San Diego, CA, USA). For combination therapy, the coefficient drug interaction (CDI) was calculated as CDI = AB/(A × B), where AB was the absorbance ratio of the combination groups to control group, while A and B were the ratio of the single-agent group to control group. CDI value < 1, =1 or >1 indicates that the drugs are synergistic, additive or antagonistic, respectively.

### 2.6. Western Blotting

Cell lines were seeded with a confluence of 1.5 × 10^5^ cells/mL on a 100 mm plate and allowed to grow for 24 h. Afterwards, cells were treated with different compounds for 24 h: CQ (150 µM), 3-MA (10 mM) and LAI-1 (10, 15 and 20 µM). A second experiment analyzing the effect of 10 µM of LAI-1 at different time points along 48 h was performed. Protein extracts were obtained from cells through the addition of RIPA lysis buffer (0.1% SDS, 1% NP-40 and 0.5% sodium deoxycholate in PBS) with 40 mM β-glycerophosphate, 50 mM sodium fluoride, 1 mM sodium orthovanadate, 1 mM phenylmethylsulfonyl fluoride and a serine and cysteine protease inhibitor cocktail (Roche, Manheim, Germany). Protein quantification was performed following the BCA protein assay kit (Pierce, Rockford, IL, USA). After that, 30 μg of protein extracts were separated with 15% SDS-polyacrylamide gel electrophoresis and transferred to a PVDF membrane (Bio-Rad, Hercules, CA, USA). Membranes were blocked for 1 h in 5% milk in TBS-Tween (50 mM Tris–HCl pH 7.5, 150 mM NaCl, 0.1% Tween-20). Then, membranes were incubated overnight at 4 °C with the following primary antibodies: anti-LC3 (Cat#PM036), anti-p62/SQSTM1 (Cat#PM045) from MBL International Corporation (Woburn, MA, USA), anti-p53 (6243) and anti-procaspase-3 (sc-7272) from Santa Cruz Biotechnology (Dallas, TX, USA), anti-p21 (2947), anti-Akt (9272), anti-phospho-Akt (S473) (9271), anti-mTOR (2972) and anti-GAPDH (2118) from Cell Signaling Technology (Danvers, MA, USA) and anti-phospho-mTOR (S2448) (2056) from Signalway Antibody (Greenbelt, MD, USA). Antibody binding was detected using goat antimouse IgG-HRP (Cat#sc-2005) and goat antirabbit IgG-HRP (Cat#sc-2004) secondary antibodies with ECL detection kit (GE Healthcare, Chicago, IL, USA). GAPDH was used as gel loading control. Images were captured on an ImageQuantTM LAS 500 (GE Healthcare) and band densitometries were retrieved using the software ImageJ v1.52 (National Institutes of Health, Bethesda, MD, USA).

### 2.7. Flow Cytometry

After seeding the different cell lines in 6-well plates (1.35 × 10^5^ cells/mL), cells were incubated for an additional 24 h. Later, cells were treated for 24 h with different concentrations of LAI-1 (10, 15, 20 µM). After that, adherent and non-adherent cells were collected to be stained with cell death Annexin-V APC/Sytox Green kit (Beckton Dickinson, Franklin Lakes, NJ, USA) following manufacturer instructions. Flow cytometry analysis was completed using a FACS Canto II^TM^ and Diva software (Beckton Dickinson). The percentage of early apoptotic (Annexin-V+; Sytox-) cells and late apoptotic or necrotic (Annexin-V+; Sytox+) cells was assessed. At least 10,000 events were obtained from each condition.

### 2.8. Live-Cell Imaging

The different cell lines were seeded (2 × 10^4^ cells/well) in an 8-well sterile μ-Slide (Ibidi, Gräfelfing, Germany). Cells were treated with LAI-1 at different concentrations and for different times depending on the experiment. For lambda-scan analysis, cells were treated for 3 h with 10 µM of LAI-1 and emission fluorescent intensity every λ = 10 nm between 405 nm and 720 nm was captured after the excitation with each 405, 488, 561 and 633 nm lasers. To localize lysosomes inside the cell, Lysotracker Green (Molecular Probes, OR, USA) at 500 nM was preincubated for 1 h. To analyze the nucleus morphology of live and dead cells, a combination of propidium iodide (0.5 µg/mL, 1.5 h) with Hoechst 33,342 (1 µg/mL, 0.5 h) staining was performed. Images were captured using a Carl Zeiss LSM 880 spectral confocal laser scanning microscope (Carl Zeiss Microscopy GmbH, Jena, Germany) and processed with ZEN 2 blue edition software (Zeiss). The colocalization analysis was performed using JACOP plugin from ImageJ software. Representative images from three independent experiments are shown.

### 2.9. Immunofluorescence

A549 cells (2 × 10^5^ cells/mL) were harvested on glass coverslips for 24 h in a 12-well plate. Next day, cells were treated with CQ at 50 µM or LAI-1 at 15 µM at different time points (4 h and 24 h). Lower concentrations of CQ were used in this experiment to obtain enough cells attached on the coverslips after treatment. Once cells were treated, cells were fixed for 20 min with 4% paraformaldehyde solution. After three washes with PBS, fixed cells were permeabilized with 50 µg/mL digitonin for 10 min and then blocked with 5% normal goat serum and 2% bovine albumin serum in PBS for 1 h. Afterwards, cells were incubated overnight at 4 °C with rabbit anti-LC3 (1:500 dilution, Cat#PM036) or rabbit anti-p62/SQSTM1 (1:500 dilution, Cat#PM045) from MBL International Corporation for phagosome characterization, and with mouse anti-LAMP1 (1:200 dilution, Cat#14-1079-80) from eBioscience (Santa Clara, CA, USA) for lysosome identification. Next day, cells were incubated with secondary antibodies, donkey antirabbit 488 nm (Cat#A-21206, Invitrogen, Waltham, MA, USA) and donkey antimouse 647 nm (Cat#A21236, Invitrogen) at 1:750 in the dark for 1 h. After three washes with PBS, cells were placed on slides using DAPI Fluoromount-G mounting medium (Thermo Fisher), which also allowed us to stain the nucleus. Images were captured using Carl Zeiss LSM 880 spectral confocal laser scanning microscope (Zeiss) and processed with ZEN 2 blue edition software (Zeiss). The fluorescence intensity was measured with ImageJ software and representative images from three independent experiments are shown.

### 2.10. Acridine Orange Staining

A549 cells (1.5 × 10^5^ cells/mL) were cultured in a 12-well plate on glass coverslips for 24 h. The next day, cells were treated with different concentrations of LAI-1 or CQ for 1 h. After two washes with PBS, cells were stained with an acridine orange (AO) solution at 5 µg/mL for 30 min at room temperature in darkness. Then, cells were washed three times with PBS/10% FBS, and the coverslips with stained cells were observed with NIKON eclipse E800 microscope using a 330/380 nm filter (Nikon Europe BV, Badhoevedorp, The Netherlands).

### 2.11. Intracellular pH Determination

A549 cells were seeded in a 96-well black well plate with clear bottom (1 × 10^4^ cells/well). After 24 h, cells were treated for 1 h with increasing concentrations of LAI-1. Intracellular pH determination was performed with pH Rodo Red AM staining kit from Molecular Probes. Briefly, cells were washed with a HEPES-based buffer (20 mM, pH 7.4) and incubated with pH Rodo Red AM staining for 30 min at 37 °C. After two additional washes, the emission at 590 nm after excitation at 550 nm was detected with FLUOstar OPTIMA (BMG LabTech, Ortenberg, Germany). Quantification was applied by extrapolating test values to an Intracellular pH Calibration Curve kit (Molecular Probes).

### 2.12. Statistical Analysis

Collected data from at least three independent replicates of the different techniques were statistically analyzed using GraphPad Prism 8.0 software. One-way ANOVA with post hoc Dunnett’s test for multiple comparison was carried out to compare different groups. Statistical differences were considered when *p* < 0.05.

## 3. Results

### 3.1. LAI-1 Synthesis and Anion Transport Capacity Evaluation

**Figure 1:**
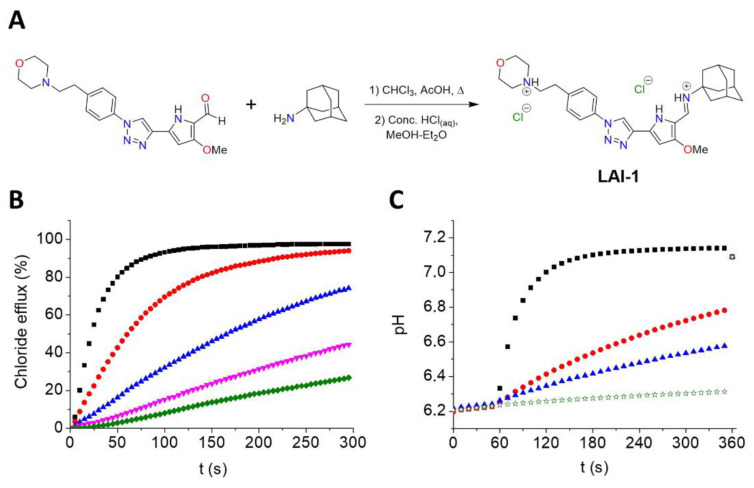
(**A**) Synthesis of LAI-1. (**B**) Transport experiments in POPC liposomes: chloride efflux promoted by compound LAI-1 (15 µM, black; 5 µM, red; 1.5 µM, blue; 0.5 µM, pink; 0.15 µM, green) in unilamellar POPC vesicles. Vesicles, loaded with a NaCl solution (451 mM NaCl and 20 mM NaH_2_PO_4_, pH 7.2), were immersed in a Na_2_SO_4_ solution (150 mM Na_2_SO_4_, 40 mM NaHCO_3_ and 20 mM NaH_2_PO_4_, pH 7.2). (**C**) Transport experiments in POPC liposomes: variation of pH upon addition of compound LAI-1 (50 nM, black; 5 nM, red; 2.5 nM, blue; blank, green) to 7:3 POPC:cholesterol vesicles (0.5 mM POPC). Vesicles, loaded with a NaNO_3_ buffered aqueous solution (126.2 mM NaNO_3_, 10 mM NaH_2_PO_4_, 1 mM HPTS, pH 6.2), were suspended in a NaNO_3_ buffered aqueous solution (126.2 mM NaNO_3_, 10 mM NaH_2_PO_4_, pH 7.5) just before starting the measurements. At t = 60 s, the compound (or the blank, DMSO, 6.25 μL) was added, and at t = 360 s, a detergent (Triton-X, 20% dispersion in water, 20 μL) was added.

LAI-1 was prepared through the acid-catalyzed condensation of 1-adamantylamine and the appropriate aldehyde (Figure 1A). The presence of morpholine aimed to include a basic group in the molecule, which would allow the accumulation of this compound in acidic compartments, such as lysosomes. This compound has an anion binding pocket, in which three hydrogen bond donors (imine and pyrrole N-H as well as polarized triazole C-H) can cooperatively interact with anions, forming a supramolecular complex capable of diffusing across the lipid bilayer.

The ability of LAI-1 to facilitate the transport of anions was first explored in model POPC liposomes (Figure 1B and Supplementary Materials Figures S13–S16). This compound was shown to efficiently promote the exchange of chloride, nitrate and bicarbonate across the lipid bilayer using a chloride-selective electrode. This activity was concentration-dependent, and the concentration needed to elicit the transport of 50% encapsulated chloride was determined as the EC_50_ parameter (Table S1). The higher hydrophilicity of bicarbonate compared to that of nitrate caused the bicarbonate anion to be more difficult to extract into the membrane; thus, a higher EC_50_ concentration was determined for this anion. A detergent effect or unspecific chloride leakage was ruled out by the inability of LAI-1 to release encapsulated carboxyfluorescein (see Supplementary Materials Figures S17–S19). Using the pH-sensitive dye HPTS (Pyranine), the ability of LAI-1 to discharge pH gradients across liposomal membranes was also explored. Vesicles encapsulating a pH 6.2 solution were dispersed in isotonic pH 7.5 media. Monitoring the internal pH of the liposomes upon the addition of LAI-1, an increase in the pH was observed. This increase was again concentration-dependent and supported the activity of LAI-1 as an active transporter capable of inducing the basification of acidic microenvironments as a result of its anionophoric activity (Figure 1C and Supplementary Materials Figures S20 and S21).

### 3.2. LAI-1 Is Specifically Internalized to the Lysosomes

To corroborate that LAI-1 enters the cell and specifically translocates to the lysosomal membrane, a colocalization analysis using confocal microscopy was performed. First, to evaluate the autofluorescence of LAI-1, a lambda scan analysis was carried out from 405 nm to 720 nm (Supplementary Materials Figure S12). The excitation using a 561 nm laser was finally chosen due to good specificity and lower background.

**Figure 2:**
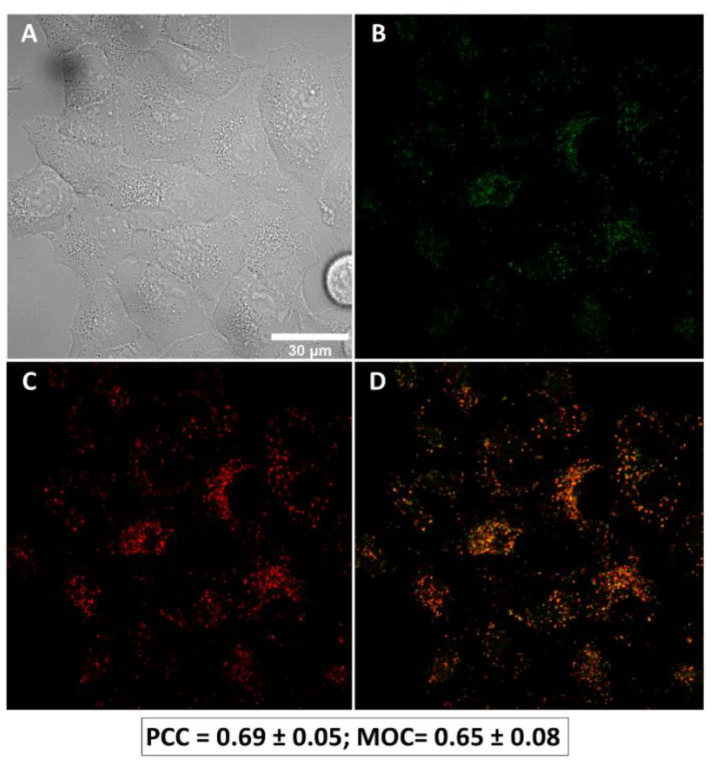
LAI-1 and lysosomes subcellular colocalization in A549 cell line. Live cell imaging fluorescence microscopy was performed. (**A**) Differential interference contrast of A549 cells. (**B**) Lysotracker Green labelling lysosomes (green channel). (**C**) A total of 10 µM of LAI-1 treatment after 3 h (red channel). (**D**) Merged channel showing lysosomes and LAI-1 colocalization (orange). Images are representative of three independent experiments. Scale bar 30 µm. Colocalization between Lysotracker and LAI-1 was quantified using Pearson’s correlation coefficient (PCC) and Mander’s overlap coefficient (MOC).

To analyze the subcellular localization of LAI-1, A549 cells were treated with 10 µM of LAI-1 for 3 h and with the Lysotracker Green lysosomal tracker for 1 h. As observed in Figure 2, a significant colocalization was detected between LAI-1 (red) and lysosomes (green). Colocalization ratios were calculated using Mander’s overlap coefficient (0.65 ± 0.08) and Pearson’s correlation coefficient (0.69 ± 0.05), both showing high values. Therefore, we confirmed that our synthetic anionophore was reaching its target organelle, the lysosome.

### 3.3. Autophagy Inhibitors Affect Lung Cancer Cell Viability

**Figure 3:**
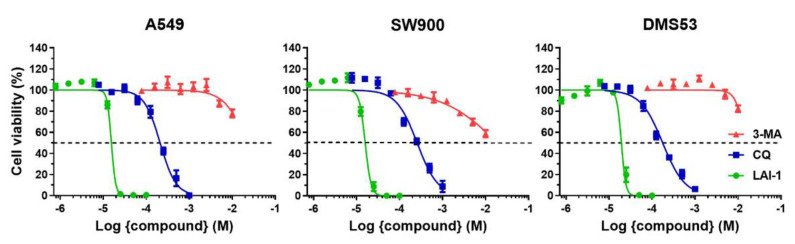
Cell viability dose–response curves, evaluated with MTT assay, for all cell lines (A549, SW900 and DMS53) after 24 h incubation with chloroquine (CQ), 3-Methyladenine (3-MA) and LAI-1 treatment at different concentrations. Each point represents the mean value ± SD.

LAI-1 effects on cell viability were assessed in three lung cancer cell lines (A549, SW900 and DMS53) representative of the three major histological subtypes, adenocarcinoma, squamous cell lung carcinoma and non-small cell lung cancer, respectively (Figure 3). Cell viability was evaluated with the MTT assay after 24 h of treatment. The inhibitory concentration (IC) IC_25_, IC_50_ and IC_75_ values of this compound for the three cancer cell lines are shown in Table 1. No significant differences were observed among them, despite their different genetic background, although A549 was the cell line with the lowest IC_50_ (15.47 ± 0.79 µM). Furthermore, cells were also treated with two well-characterized autophagy inhibitors, chloroquine (CQ) (Table S2) and 3-methyladenine (3-MA) (Table S3). LAI-1 was the most potent anticancer compound decreasing cell viability, compared to CQ and 3-MA, both showing higher IC_50_ values (Figure 3). These results indicated that the LAI-1 compound presented a greater detrimental effect on cell viability compared to other well-known autophagy inhibitors.

### 3.4. LAI-1 Is a Late-Phase Autophagy Inhibitor

**Figure 4:**
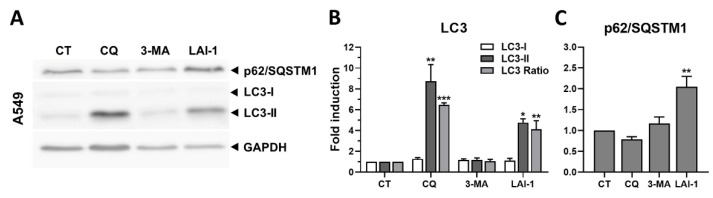
Autophagy markers after treatment with different autophagy inhibitors. (**A**) Western blot showing autophagy-related proteins LC3 and p62/SQSTM1 after 24 h with chloroquine (CQ, 150 µM), 3-Methyladenine (3-MA, 10 mM) and LAI-1 (10 µM) treatment in A549 cells. (**B**) LC3-I and LC3-II protein levels and their LC3-II/I ratios. (**C**) p62/SQSTM1 protein expression. Protein expression was normalized using GAPDH as loading control. Fold induction against control group (CT) was calculated. Figure shows mean ± SEM. Statistical differences against CT are shown as *** *p* < 0.001, ** *p* < 0.01 and * *p* < 0.05. The whole western blot figures are showed in Figure S24.

To analyze the molecular effects induced after inhibiting autophagy at the initial or the final stage of the process, the well-known autophagy inhibitors 3-MA and CQ were used. They were used to compare them with the effects induced by LAI-1 and elucidate whether this compound was an early- or late-stage autophagy inhibitor. A Western blot analysis was performed to detect autophagy-related proteins LC3 and p62/SQSTM1 after treating A549 cells for 24 h with CQ (150 µM), 3-MA (10 mM) and LAI-1 (10 µM) (Figure 4). A low dose of LAI-1 was used to elucidate whether autophagy was being modified by LAI-1 before affecting cellular viability. Our results showed a significant increase in the LC3 II/I ratio in A549 cells after the LAI-1 treatment, mainly due to an increase in the LC3-II protein, indicating autophagosome accumulation. While the LC3-II and LC3-II/I ratio were also strongly increased after the CQ treatment, a late-phase autophagy inhibitor, no effect on LC3-I and LC3-II was observed after the 3-MA treatment, an initial-stage autophagy inhibitor. Hence, LAI-1 behaved similarly to CQ, which inhibited autophagy at the late-phase stage. This autophagosomes accumulation can be induced by autophagy activation and/or blockage. To elucidate whether autophagy blockage was occurring, p62/SQSTM1 expression was also evaluated, since this protein degrades at the end of the autophagy process. p62/SQSTM1 protein levels significantly increased after the LAI-1 treatment, indicating that the protein was not being degraded; thus, autophagy was effectively induced by this compound (Figure 4). In contrast, no accumulation of p62/SQSTM1 was observed after the 3-MA or CQ treatments. Considering these results, CQ (150 µM) induced autophagy blockage, triggering a higher autophagosome accumulation than the other inhibitors; however, LAI-1 was also able to effectively block late-stage autophagy, showing an autophagosome accumulation at a lower concentration (10 µM).

### 3.5. LAI-1 Effectively and Rapidly Blocks Autophagy in Lung Cancer Cells

**Figure 5:**
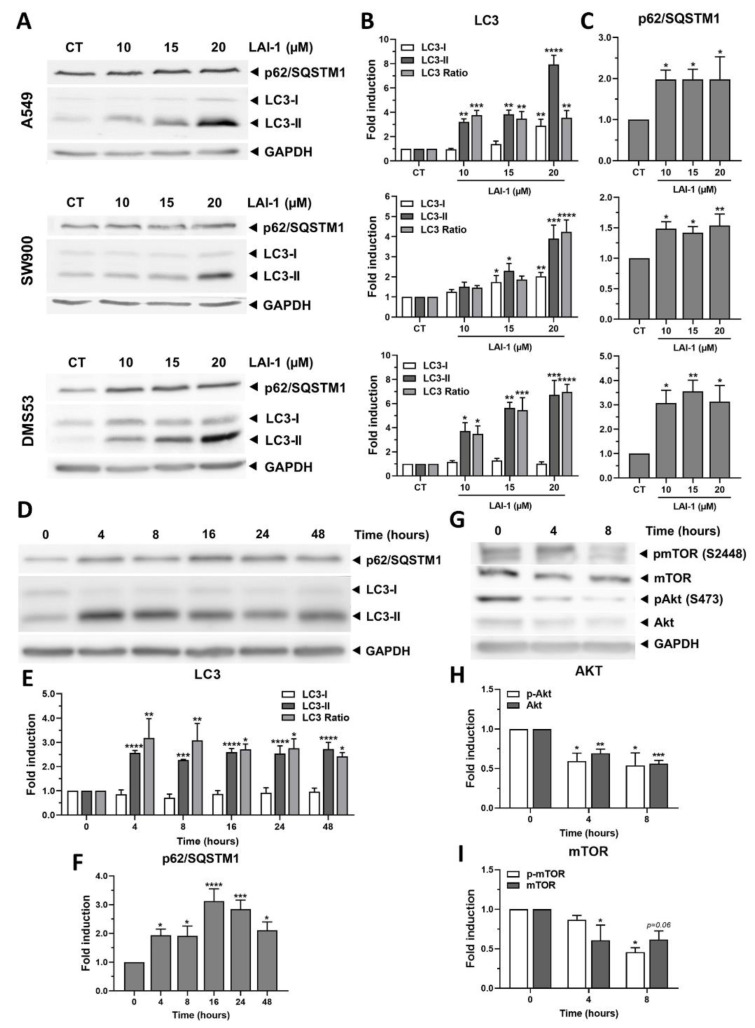
Autophagy modulation after LAI-1 treatment. (**A**) Dose–response Western blot showing autophagy-related proteins LC3 and p62/SQSTM1 after 24 h of LAI-1 treatment at different concentrations (10, 15 and 20 µM) in A549, SW900 and DMS53 cell lines. (**B**) LC3-I and LC3-II protein levels and their LC3-II/I ratios after dose–response assay. (**C**) p62/SQSTM1 protein expression after dose–response experiment. (**D**) Time-course Western blot showing autophagy-related proteins LC3 and p62/SQSTM1 after treating A549 cells with LAI-1 (10 µM) at different time points (0, 4, 8, 16, 24, 48 h). (**E**) LC3-I and LC3-II protein levels and their LC3-II/I ratios after time-course experiment. (**F**) p62/SQSTM1 protein expression after time-course assay. (**G**) Time-course Western blot showing autophagy activation proteins Akt and mTOR after treating A549 cells with LAI-1 (10 µM) at different time points (0, 4, 8 h). (**H**) Akt and phospho-Akt at S473 (pAkt) protein levels after time-course experiment. (**I**) mTOR and phospho-mTOR at S2778 (pmTOR) protein expression after time-course assay. Protein expression was normalized using GAPDH as loading control. Fold induction against control group (CT) was calculated. Figure shows mean ± SEM. Statistical differences against CT are shown as * *p* < 0.05, ** *p* < 0.01, *** *p* < 0.001 and **** *p* < 0.0001. The whole western blot figures are showed in Figures S25–S29.

In order to more deeply analyze how LAI-1 induces autophagy inhibition, different lung cancer cell lines were treated at different drug concentrations (10, 15 and 20 μM) for 24 h. A Western blot analysis was performed detecting the expression of autophagy-related proteins LC3 and p62/SQSTM1 (Figure 5A). Our results showed a dose-dependent increase in LC3-II in all cell lines, especially A549 and DMS53, displaying its highest effect at 20 µM (Figure 5B). As a consequence, an increase in the LC3-II/I ratio was also observed in all cell lines, indicating autophagosome accumulation. On the other hand, p62/SQSTM1 protein expression was significantly increased in all cell lines after treatment with LAI-1 in all the tested concentrations, suggesting that this protein was not being degraded due to the autophagy blockage (Figure 5C). Altogether, LAI-1 potently blocked autophagy at concentrations higher than 15 μM, as it significantly increased LC3-II/I and p62/SQSTM1 levels in all lung cancer cell lines, without relevant differences.

To further characterize LAI-1 effects on autophagy, a time-course assay after treatment with 10 µM of LAI-1 was performed on A549 cells to analyze the time with the highest effect on autophagy modulation (Figure 5D). Our quantifications revealed that LC3-II protein expression was significantly increased after 4 h, maintaining similar protein levels along 48 h (Figure 5E). The enhanced expression of LC3-II accompanied with no changes in LC3-I along 24 h was translated into the significantly increased LC3-II/I ratio in all the time points studied. In the case of p62/SQSTM1, even after 4 h of treatment, its protein expression was enhanced, showing its maximum accumulation between 16 h and 24 h (Figure 5F). These results indicated that the maximum autophagy blockage induced by LAI-1 occurred as early as 4 h after treatment, and this effect was maintained for at least 48 h.

Finally, to analyze whether LAI-1 was also modulating autophagy induction, the negative autophagy regulator PI3K/mTOR pathway was studied. AKT and mTOR phosphorylation were assessed at short time points of 4 and 8 h (Figure 5G). As observed in Figure 5H,I, the LAI-1 treatment induced a decrease in AKT as well as in mTOR phosphorylation levels, suggesting that autophagy was being triggered in these conditions. Altogether, these results showed that the LAI-1 treatment was able to enhance autophagy activation by diminishing mTOR phosphorylation levels, while also blocking the autophagy flux at a later stage.

### 3.6. LAI-1 Blocks Autophagosomes with Lysosomes Fusion

Our previous results showed an increase in p62/SQSTM1 and LC3 protein expression with Western Blot analysis after the CQ and LAI-1 treatments, suggesting an autophagy blockage. To better understand how LAI-1 modulated the autophagy process, we analyzed the localization of p62/SQSTM1 and LC3 autophagosomal markers in combination with LAMP-1, a lysosomal protein, to examine autophagosome accumulation and the fusion of autophagosomes with lysosomes. To perform these experiments, LAI-1 (15 µM) and lower concentrations of CQ (50 µM), to prevent cell detachment, were used to obtain an adequate number of cells attached to the plate.

**Figure 6:**
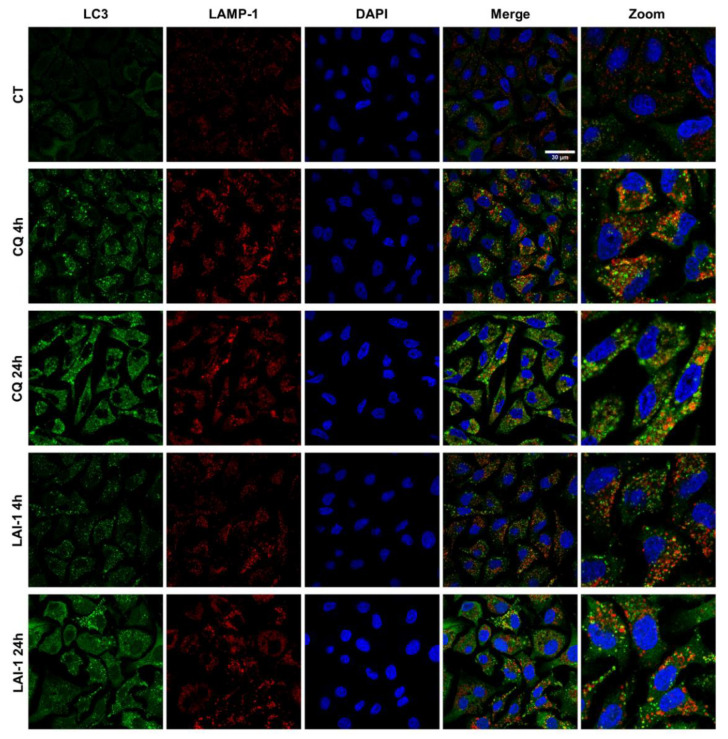
Subcellular localization of LC3 and LAMP-1. A549 cells were harvested for 24 h on coverslips and then treated with LAI-1 (15 µM) and chloroquine (CQ, 50 µM) for different times. The fusion between autophagosomes, marked with LC3 (green), and lysosomes, marked with LAMP-1 (red), was studied in merged images. Zoomed images were used to better observe this process. The localization and intensity of LC3 staining were also analyzed. DAPI (blue) staining was used for nuclear localization. Images are representative of three independent experiments. Scale bar 30 µm.

In Figure 6, representative images of LC3 expression (green) in combination with LAMP-1 (red) in A549 cells treated for 4 h and 24 h with CQ and LAI-1 are shown. Initially, our results showed that control cells expressed very few LC3 protein levels, presenting small dots in some cells, suggesting a very low autophagy basal level. In contrast, cells after the CQ treatment significantly increased the expression of LC3, showing a higher green signal than control cells in both time points with a maximum effect after 24 h (5.22 ± 0.97-fold increase, Supplementary Materials Table S4). The zoomed images after these conditions showed bigger green dots (LC3) that poorly colocalized with the red dots (LAMP-1), indicating that lysosome and autophagosome fusion did not occur. In this cell line, the LAI-1 treatment also significantly increased the signal of LC3 compared to control cells, but lower than the CQ treatment. However, green staining also revealed an accumulation in big dots (autophagosomes), especially after 24 h. Moreover, as observed in the amplified images, LAI-1-treated cells did not present colocalization between the green and red dots either. In fact, it showed a lower colocalization than the CQ treatment, indicating the blockage of autophagosomes and lysosome fusion, as well.

Similar results were observed in Supplementary Materials Figure S22, where representative images of the p62/SQSTM1 protein (green) in combination with LAMP-1 (red) in A549 cells after 4 h and 24 h treatment with CQ and LAI-1 are presented. Cells treated with LAI-1 showed a significant increase in p62/SQSTM1 in comparison with the control group (2.63 ± 0.77 and 4.23 ± 0.73-fold increase at 4 h and 24 h, respectively, Supplementary Materials, Table S4). In both time points, the expression of p62/SQSTM1 in the cytosol was enhanced, showing small and more bright dots than the control group, especially after 4 h of treatment. Similarly to the LC3 analysis, neither the LAI-1 treatment nor the CQ treatment displayed any colocalization between the green and red dots, corroborating the inhibition of the fusion among autophagosomes and lysosomes. Altogether, these results confirmed the increasing expression of p62/SQSTM1 and LC3 after 15 µM of the LAI-1 treatment observed previously with the Western blot analysis. Moreover, our results showed that there was a blockage of autophagy after the LAI-1 and CQ treatments caused by the non-fusion of mature autophagosomes (LC3 and p62/SQSTM1 positive cells) with lysosomes (LAMP-1).

### 3.7. LAI-1 Treatment Modifies Lysosomal and Cytosolic pH

**Figure 7:**
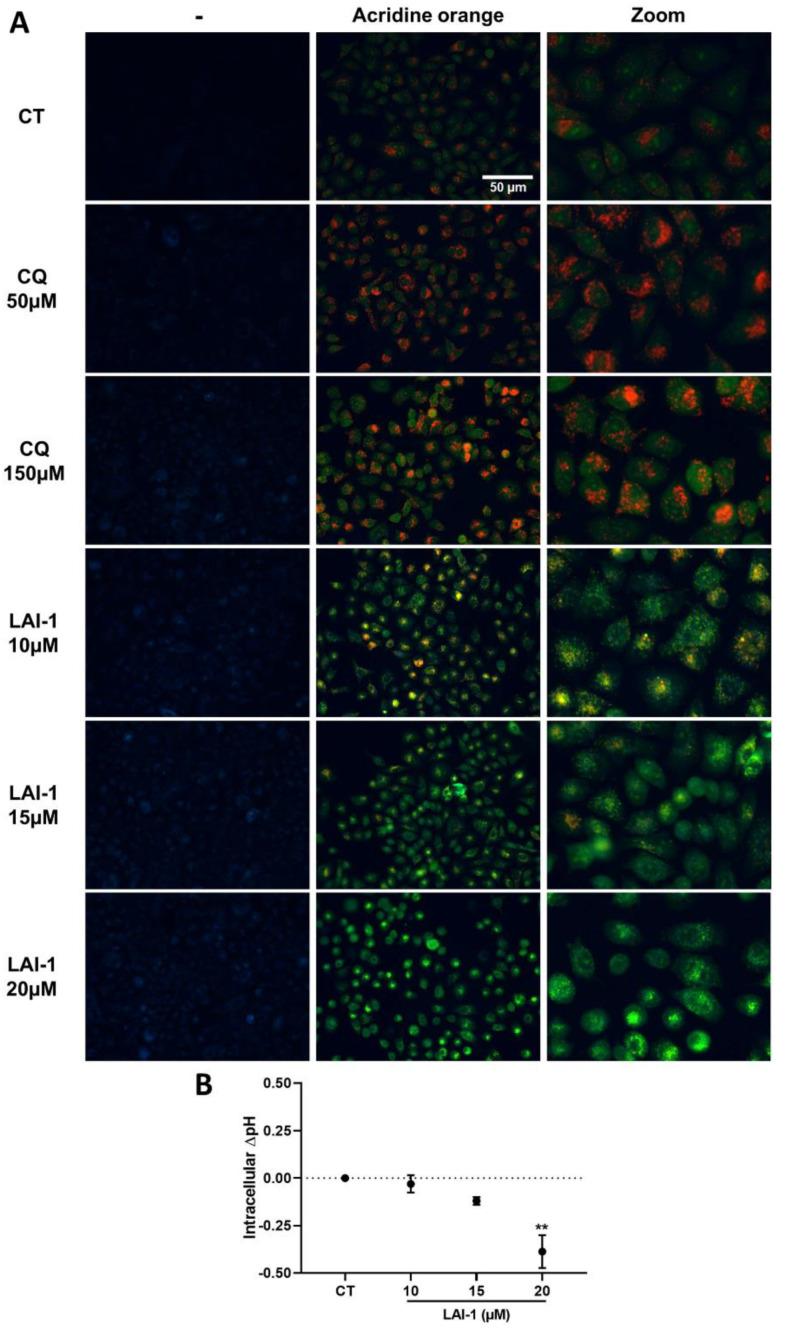
Lysosomal and intracellular pH modifications using LAI-1 treatment. (**A**) A549 cells were treated for 1 h with LAI-1 or chloroquine (CQ) at different concentrations. After that, acridine orange staining (5 µg/mL) was performed for 30 min at room temperature. Images are representative of three independent experiments. Scale bar 50 µm. (**B**) Intracellular pH measurement in A549 cells treated with different LAI-1 concentrations for 1 h. Staining with pH Rodo Red AM staining kit and a calibration curve was conducted to quantify intracellular pH. Figure shows mean ± SEM. Statistical differences against control group (CT) are shown as ** *p* < 0.01.

To confirm the capacity of the LAI-1 compound to act as an anionophore across the lysosomal membrane, the acridine orange (AO) staining procedure was used. Due to the weak emission of LAI-1 in the blue spectrum (Supplementary Materials, Figure S12), images without AO staining were taken to elucidate a possible interference of this drug. The images on the left column of Figure 7A showed that different concentrations of LAI-1 displayed a weak interference in blue fluorescence that did not alter the subsequent analysis. After AO staining, cells showed a green emission in the nucleus and other parts of the cell in all conditions, caused mainly by AO affinity to nucleic acids. In contrast, the orange emission of AO observed in dots in the cytosol is caused by the protonation of this compound, which occurs in acidic environments, such as lysosomes. This orange emission was seen in our control group and was enhanced after 1 h treatment with both CQ concentrations (Figure 7A). In contrast, our results revealed a significant decrease in the orange emission after 1 h of LAI-1 treatment in a dose-dependent manner in comparison with the control group (Figure 7A), indicating lysosomal deacidification. The lysosomal pH modifications caused by LAI-1 may have contributed to the blockage of autophagy observed in our previous results and p62/SQSTM1 accumulation due to lysosomal dysfunction. Complementary to lysosomal deacidification, intracellular pH was quantified after increasing the concentrations of LAI-1. Our results showed a decrease in the cytosolic pH after 1 h of LAI-1 treatment, reaching statistical significance at 20 µM (Δ = −0.4) (Figure 7B). Altogether, these results supported the anionophoric activity of LAI-1 across lysosomal membranes and indicated that LAI-1 provoked an increase in the pH of lysosomes, which may impede the correct function of lysosomal hydrolases, as well as induce an acidification of the cytoplasm.

### 3.8. Cell Death Induction after LAI-1 Treatment

**Figure 8:**
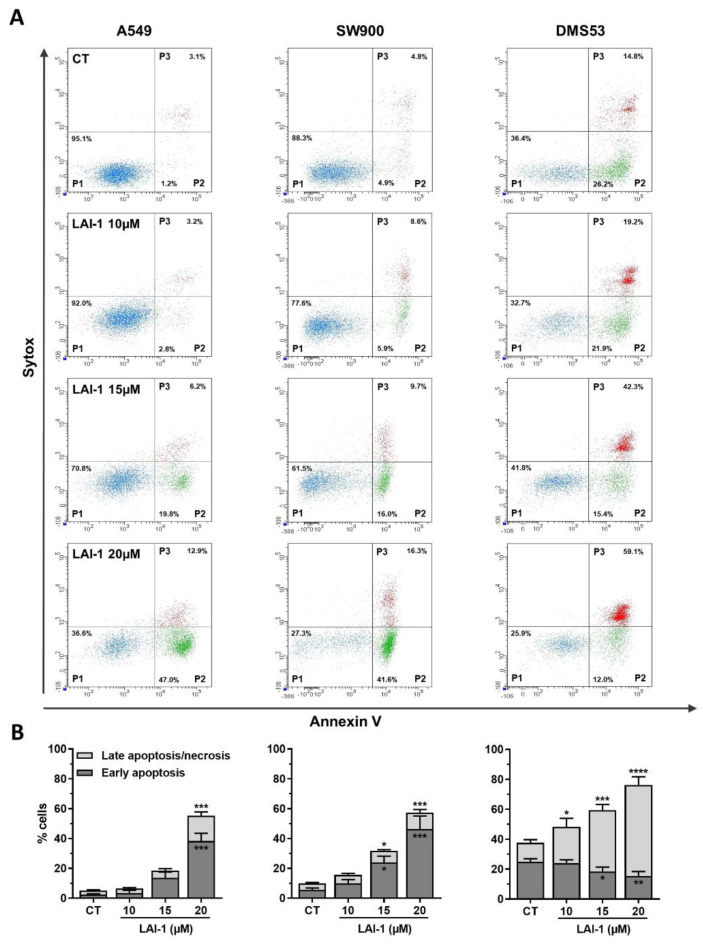
Cell death characterization using flow cytometry. The different cell lines (A549, SW900 and DMS53) were treated for 24 h with different LAI-1 concentrations. Cells were stained with Annexin-V APC/ Sytox Green kit. Early apoptotic cells were defined as Annexin-positive and Sytox-negative, whereas late apoptotic or necrotic cells were defined as Annexin- and Sytox-positive. (**A**) Representative plots. (**B**) Quantification of % of cells in early apoptosis and in late apoptosis or necrosis after different treatment. Statistical differences against control group (CT) are shown as * *p* < 0.05, ** *p* < 0.01, *** *p* < 0.001 and **** *p* < 0.0001.

To identify whether the decrease in cell viability was due to apoptosis or necrosis, a cell death analysis using flow cytometry was performed. A549, SW900 and DMS53 cell lines were treated with LAI-1 at different concentrations (10, 15 and 20 μM) for 24 h (Figure 8A). Our results showed that no significant cell death was observed on A549 and SW900 cells after 10 μM of LAI-1, compared to the control group (Figure 8B). However, early and late apoptotic/necrotic cells, especially early apoptosis, were significantly increased in a concentration-dependent manner after ≥15 μM LAI-1 in both cell lines, indicative of induced apoptotic cell death. On the other hand, DMS53 late apoptotic/necrotic cells significantly increased at all concentrations in a dose-dependent manner, while early apoptosis decreased as the concentration rose, suggesting that most of these cells may have died from necrosis rather than apoptosis. This result corroborated the morphological study with the double staining with propidium iodide and Hoechst dye in DMS53 LAI-1-treated cells, which revealed that most of the doubled-stained cells presented either cell membrane swelling or intracellular leakage, typical of the necrotic process (Supplementary Materials, Figure S23). Altogether, these results indicated an increased cell death mainly after concentrations ≥15 µM of LAI-1 in all cell lines.

### 3.9. LAI-1 Induces Cell Death but Not Cell Cycle Arrest

**Figure 9:**
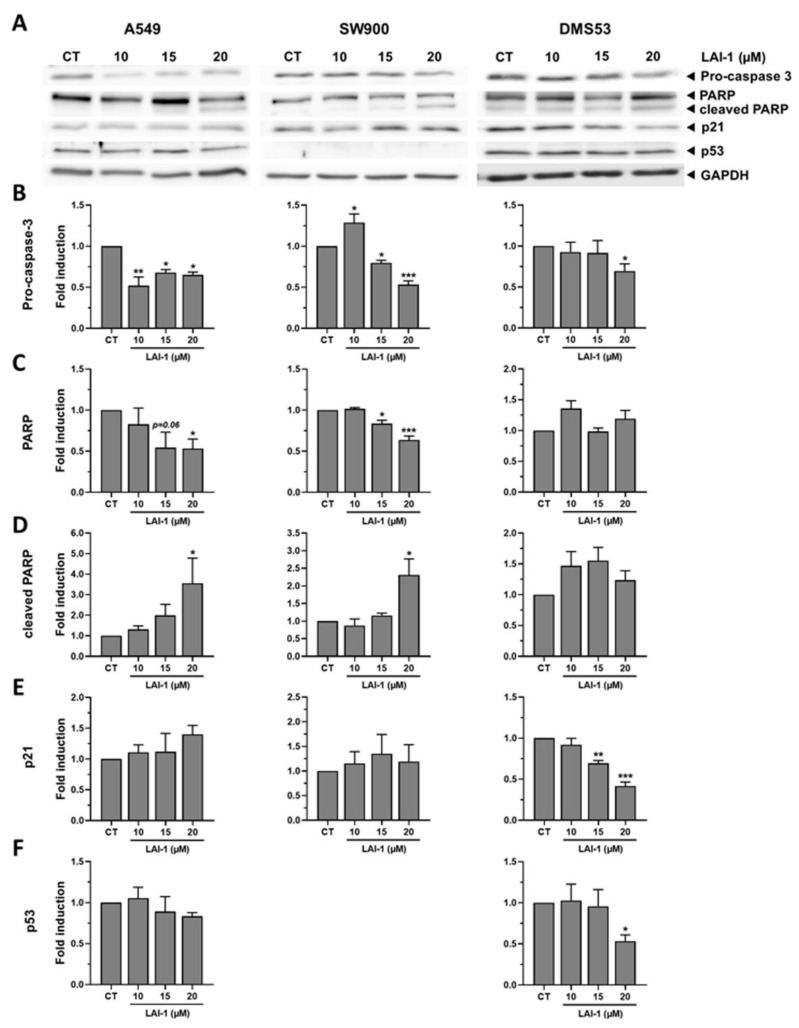
Expression of apoptotic and cell-cycle related proteins. The different cell lines (A549, SW900 and DMS53) were treated for 24 h with different LAI-1 concentrations and several proteins were assessed with Western blot (**A**). Quantification of procaspase 3 (**B**), PARP (**C**), cleaved-PARP (**D**), p21 (**E**) and p53 (**F**) protein expression after Western blot analysis was performed using GAPDH expression as loading control. Fold induction against control group (CT) was calculated. Figures show mean ± SEM. Statistical differences against CT are shown as * *p* < 0.05, ** *p* < 0.01 and *** *p* < 0.001. The whole western blot figures are showed in Figures S30–S32.

In order to further study the molecular effects induced by the LAI-1 treatment, proteins related to apoptosis (procaspase 3, PARP) and cell cycle arrest (p21 and p53) were assessed with a Western blot analysis in cancer cell lines after treatment with increasing concentrations of LAI-1 for 24 h (Figure 9).

Our results showed that apoptosis was activated after the LAI-1 treatment in A549 and SW900 cells, since procaspase 3 levels decreased and the cleaved PARP increased in a concentration-dependent manner, indicative of caspase activation (Figure 9B,D). However, DMS53 cells showed only a slight caspase activation at 20 µM and no significant PARP cleavage, supporting the previous result that most of the observed cell death was not due to apoptosis but to necrosis. The differences observed in the type of cell death induced by LAI-1 may be due to differences in the genetic background of the three cell lines. On the other hand, no significant increase was observed in the cell cycle arrest markers p21 and p53 (Figure 9E,F). Therefore, our results confirmed that apoptosis and necrosis were activated in all cell lines after the LAI-1 treatment, whereas no significant effect on cell cycle arrest was observed.

### 3.10. LAI-1 Sensitizes Lung Cancer Cells to Cisplatin Treatment

**Figure 10:**
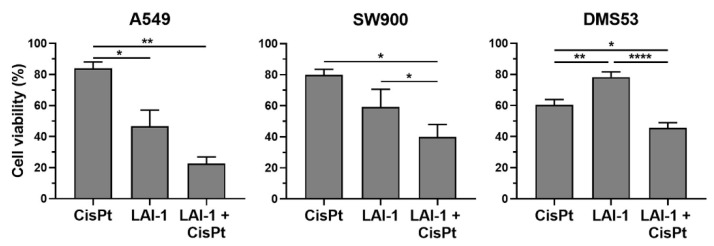
Cell viability, evaluated with MTT assay, for A549, SW900 and DMS53 lung cancer cell lines after 24 h incubation with cisplatin (CisPt, 40 µM), LAI-1 (15 µM) and the combination. Figure shows mean ± SEM. Statistical differences are shown as **** *p* < 0.0001 ** *p* < 0.01 and * *p* < 0.05.

Finally, since autophagy inhibitors in combination with chemotherapeutics are, nowadays, being tested in several clinical trials for tumor treatment sensitization, LAI-1 was administered to A549, SW900 and DMS53 lung cancer cells in combination with the first-line chemotherapeutic agent cisplatin (CisPt) (Figure 10). The combination of both treatments showed a significantly lower cell viability than both treatments alone, except against LAI-1 in A549 cells, indicating that LAI-1 was able to sensitize cancer cells to conventional treatments. The coefficient of drug interaction (CDI) method was used to evaluate the combined effect. The average of CDI values was 0.56 ± 0.28 for A549, 0.89 ± 0.04 for SW900 cells and 1.06 ± 0.17 for DMS53, indicating a synergistic effect (CDI < 1) in A549 and SW900 cells and an additive effect (CDI = 1) in DMS53 cells. Hence, autophagy has a cytoprotective role in these tumor cells, which can be abolished by LAI-1, contributing to sensitizing cancer cells to conventional treatments.

## 4. Discussion

Treatment resistance is responsible for most cancer relapses, being one of the leading causes of death worldwide [5]. Therefore, inhibiting the mechanisms that confer resistance to cancer cells is one of the major challenges in cancer therapy. Autophagy is a cellular catabolic process that can confer resistance to tumor cells; hence, the inhibition of autophagy is a promising therapeutic strategy for cancer treatment [20]. Here, we characterized a novel autophagy inhibitor, which specifically targets and deacidifies lysosomes, leading to an efficient late-stage autophagy blockage and to a potent cytotoxic anticancer effect in lung cancer cells.

We designed the lysosome-targeting anionophore LAI-1 by decorating a click-tambjamine derivative with a basic morpholine moiety [21]. This type of compound was recently reported by us as a bioactive anion transporter. The design was modified in order to incorporate a morpholine group in the molecule. This is a strategy successfully employed to direct small molecules, including anionophores, to lysosomes [22]. Other lysosomotropic agents, such as CQ and its analog hydroxychloroquine (HCQ), have been widely used in cancer clinical trials to inhibit autophagy [23]. However, higher micromolar concentrations are required to block autophagy, and some side effects, such as retinal toxicity, have been described [24,25]. In this study, we confirmed that lower concentrations of LAI-1 were sufficient to effectively inhibit autophagy at a similar level as higher CQ concentrations, showing more potent anticancer effects in three different lung cancer cell lines.

The LAI-1 treatment induced high levels of the autophagosomal marker LC3-II, indicating autophagosome accumulation. This could be due to either the activation or inhibition of autophagy, since their accumulation may result from the formation of new autophagosomes and/or the blockage of their degradation, hampering the recycling of the LC3-II protein [26]. However, an accumulation of p62/SQSTM1 after the LAI-1 treatment demonstrated that the cargo was not being degraded, which confirmed that the autophagy process was inhibited by the LAI-1 treatment. A similar effect was observed in LC3-II after the CQ treatment, which was also highly accumulated, corroborating previous results. Moreover, autophagosome and lysosome fusion was effectively inhibited after treatment with the LAI-1 compound, as demonstrated by a lack of colocalization in the immunofluorescence staining of LC3 or p62/SQSTM1 and LAMP-1 markers, respectively. This mechanism of action was similar to that observed in the literature for CQ, where this process was also inhibited, leading to autophagosome accumulation and the blockage of the autophagy process [27]. These facts demonstrate that LAI-1 efficiently blocked autophagy with a mechanism of action similar to that of CQ. On the other hand, squaramide-based anionophores were also shown to block autophagic flux and trigger the relocation of the LC3 autophagic marker towards the Golgi on human osteosarcoma U2OS cells [28].

The perturbation of homeostasis, through the facilitated anion transport activity displayed by anionophores, has been shown to impact cellular pH levels and deacidify acidic organelles [29]. The anion transport capacity across the cellular membranes of our novel compound was confirmed using a liposomal model and a cellular model. LAI-1 showed chloride and bicarbonate transport as well as the ability to dissipate pH gradients in liposomes. Supporting these results, LAI-1 anionophoric activity was observed across the lysosomal membrane in lung cancer cells, since acridine orange staining demonstrated an increase in lysosomal pH after 1 h upon treatment with increasing concentrations of LAI-1. An increase in lysosomal pH was also previously found after cell treatment with different anionophoric molecules, such as tambjamine analogs or obatoclax; however, these molecules also target other organelles [30,31,32]. The maintenance of an acidic environment inside lysosomes is crucial for their proper function, as the hydrolases that degrade damaged proteins or organelles are only functional at an acidic pH [33]. Moreover, the deacidification of lysosomes is related with a truncated fusion with autophagosomes, impeding the adequate autophagic flux. Our results showed no changes in lysosomal pH after the CQ treatment, in accordance with Mauthe et al. [27]. However, it should be noted that Jia et al. showed that CQ could increase lysosomal pH [34]. These differences could be explained by the use of different cell lines, the cell-line context and maybe the different concentrations reported. In addition, lysosomal deacidification is associated with lysosomal membrane permeabilization, which is related to different types of cell deaths, which could be contributing to LAI-1 cytotoxic effects [35].

Accompanying the deacidification of lysosomes, our results showed a decrease in intracellular pH after 1 h of treatment with LAI-1. Although intrinsic ionic transporters and pumps focused on maintaining intracellular pH homeostasis are present in cancer cells [36,37], the acidification of intracellular pH was also observed with other ionophores, such as tambjamine analogs [29,30]. The preservation of a differentiated internal pH is necessary for the proper function of organelles and for the function and structure of proteins. In our case, we hypothesize that high concentrations of LAI-1 at the lysosomal membrane facilitated the transport and exchange of bicarbonate and chloride ions between the cytosol and the lysosomal compartment, which could be accompanied by a proton efflux from lysosome, resulting in the observed pH variations. Moreover, intracellular pH acidification has been associated with apoptosis [29,30,38], whereas alkalinization has been related to tumor progression and resistance [37]. Therefore, the intracellular pH change could be one of the reasons of the induced cell death after the LAI-1 treatment at high concentrations and could also modulate the autophagy flux [39].

Moreover, it was also demonstrated that the LAI-1 compound presented the most potent anticancer effect in vitro among all the tested autophagy inhibitors, indicating that it not only blocked the autophagy process, but also caused a greater detrimental effect on cell viability compared with other autophagy inhibitors in three different lung cancer cell lines. All the different cell lines treated with concentrations ≥15 µM showed an increased cell death compared to the control group. The increased expression of cleaved PARP, accompanied by a decrease in procaspase 3, would indicate that the LAI-1 treatment induced apoptosis, especially in the A549 and SW900 cell lines. However, the results observing the nucleus staining with Hoechst and propidium iodide also suggested that this apoptosis occurred together with a necrotic cell death, being predominant in DMS53 cells. Similar results have been reported for ionophoric compounds, such as tambjamine analogs, prodiginines, obatoclax and salinomycin, inducing both apoptosis and also necrosis [29,31,40,41,42]. In our case, at basal levels, these cancer cells would be inducing cytoprotective autophagy, and after being blocked by our drug, it would trigger cell death. The cytoprotective role of autophagy has been extensively described, especially in advanced tumor stages [18,43]. For this reason, the use of autophagy inhibitors, such as our compound, could be a potential strategy for cancer treatment [9,17]. Moreover, it has been described that autophagy activation can induce treatment resistance in cancer cells [44]. For this reason, the combination of autophagy inhibitors with conventional treatments may be a promising therapy for cancer [19,45,46]. Actually, CQ and hydroxychloroquine have been involved in several clinical trials in combination with other chemotherapeutics (ClinicalTrials.gov; accessed on 20 May 2022). In this regard, our results also showed a more potent anticancer effect when combining LAI-1 and cisplatin in all cell lines, indicating a sensitization of lung cancer cells to this conventional treatment. However, further studies should be conducted to better study this issue.

## 5. Conclusions

Autophagy is one of the cellular mechanisms that confers treatment resistance in cancer cells. Our results showed that LAI-1 is a novel and potent late-stage autophagy inhibitor that specifically targets and deacidifies lysosomes, while acidifying the cytosol, due to its anionophoric properties. These effects induce a potent blockage of cytoprotective autophagy in cancer cells, avoiding autophagosome fusion with lysosomes, which provokes autophagosome accumulation, as well as lysosomal dysfunction. Consequently, LAI-1 induces a potent anticancer effect in vitro in lung cancer cells from different histological subtypes through apoptosis and necrosis activation. Moreover, a sensitizing effect when combined with cisplatin, a currently used chemotherapeutic agent, was also demonstrated for this compound. Altogether, LAI-1 is a novel and potent autophagy inhibitor that may have potential applications in cancer treatment and tumor sensitization.

## Figures and Tables

**Table 1:** Inhibitory concentration (IC); IC_25_, IC_50_ and IC_75_ values after LAI-1 treatment for A549, SW900 and DMS53 cell lines. Data show mean value ± SD.

LAI-1 (µM)	A549	SW900	DMS53
**IC_25_**	13.67 ± 0.82	13.27 ± 0.78	19.15 ± 2.52
**IC_50_**	15.47 ± 0.79	16.10 ± 0.93	21.81 ± 1.69
**IC_75_**	17.51 ± 0.75	18.56 ± 0.80	23.94 ± 2.03

## Data Availability

The data presented in this study are available on request from the corresponding author.

## Supplementary Materials

The following supporting information can be downloaded at: https://www.mdpi.com/article/10.3390/cancers14143387/s1, Supplementary Information File Contents: 1—Synthesis and characterization data of compound LAI-1 (Figures S1–S9). 2—Absorption and emission spectra of compound LAI-1 (Figures S10–S12). 3—Transmembrane anion transport experiments in vesicles (Table S1 and Figures S13–S21). 4—Supplementary biological results (Table S2–S4 and Figures S22 and S23).
