# Supplementary material for: A Novel Late-Stage Autophagy Inhibitor That Efficiently Targets Lysosomes Inducing Potent Cytotoxic and Sensitizing Effects in Lung Cancer
Source: Cancers (Basel). 2022 Jul 12;14(14):3387. doi: 10.3390/cancers14143387 (PMC9324127; doi:10.3390/cancers14143387)
Supplement: Supplementary file 1 [file cancers-14-03387-s001.zip › cancers-1761372-supplementary.pdf]

Supplementary Materials

A novel late-stage autophagy inhibitor that efficiently targets lysosomes inducing potent cytotoxic and sensitizing effects in lung cancer

Adrià Molero-Valenzuela[1†], Pere Fontova[2†], Daniel Alonso-Carrillo[2], Israel Carreira-Barral[2], Ana Aurora Torres[1], María García-Valverde[2], Cristina Benítez-García[1], Ricardo Pérez-Tomás[1], Roberto Quesada[2], Vanessa Soto-Cerrato[1,3*].

[1] Department of Pathology and Experimental Therapeutics, Faculty of Medicine and Health Sciences,
Universitat de Barcelona, L'Hospitalet de Llobregat 08907 Barcelona, Spain; adria.molero@vhir.org (A.M.-V.); atorrega30@alumnes.ub.edu (A.A.T.); cbenitezg16@ub.edu (C.B.-G.); rperez@ub.edu (R.P.-T.)

[2] Department of Chemistry, Universidad de Burgos, 09001 Burgos, Spain; pfontova@ubu.es (P.F.);
dacarrillo@ubu.es (D.A.-C.); icarreira@ubu.es (I.C.-B.); magaval@ubu.es (M.G.-V.); rquesada@ubu.es (R.Q.)

[3] Molecular Signalling, Oncobell Program, Institut d'Investigació Biomèdica de Bellvitge (IDIBELL),
L'Hospitalet de Llobregat, 08908, Barcelona, Spain

* Correspondence: vsoto@ub.edu

† These authors contributed equally to this work.

Contents

1. SYNTHESES AND CHARACTERIZATION DATA OF COMPOUND LAI-1 3
 1.1. General procedures and methods 3
 1.2. Precursor and intermediate 3
  1.2.1. Compound A 3
  1.2.2. Compound B 6
 1.3. Click-tambjamine 9
  1.3.1. Compound LAI-1 9
2. ABSORPTION AND EMISSION SPECTRA OF COMPOUND LAI-1 12
  2.1. Absorption spectrum in DMSO 12
  2.2. Emission spectra in DMSO 12
  2.3. Emission spectra inside cells 13
3. TRANSMEMBRANE ANION TRANSPORT EXPERIMENTS IN VESICLES 14
 3.1. Preparation of phospholipid vesicles 14
 3.2. ISE transport experiments 14
 3.3. Emission spectroscopy transport experiments 18
  3.3.1. Carboxyfluorescein-based assays 18
  3.3.2. HPTS-based assays 20

4. SUPPLEMEMENTARY BIOLOGICAL RESULTS 22
4.1. Cell viability studies 22
4.2. Blockage of autophagosomes and lysosomes fusion 23
4.3. Necrosis induction by LAI-1 on DMS53 cells 24
4.4. Original blots from Western blot images 25

1. SYNTHESES AND CHARACTERIZATION DATA OF COMPOUND LAI-1

1.1. General procedures and methods

Commercial reagents were employed as received without any further purification. ^{1}H, ^{13}C and DEPT-135 NMR spectra of the precursor, the intermediate and the final compound were recorded at 25 °C on a Varian Mercury-300 MHz spectrometer, using $CDCl_3$ or DMSO-d_6 as solvents, with their residual signals being used to reference the spectra. High-resolution mass spectra were recorded on an Agilent 6545 Q-TOF mass spectrometer coupled to a 1260 Infinity liquid chromatographer from the same brand; the ionization source employed was electrospray in its positive mode. Absorption and emission spectra were recorded on Hitachi U-3900 and F-7000 spectrophotometers, respectively.

1.2. Precursor and intermediate

1.2.1. Compound A

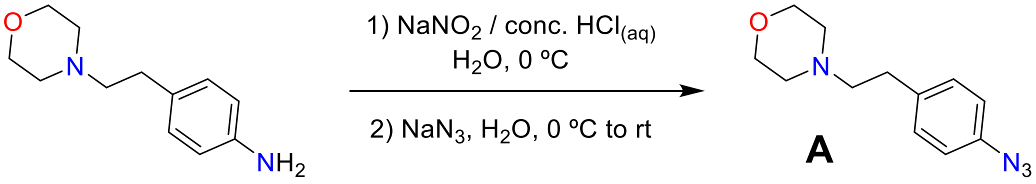

A mixture of 4-(2-morpholinoethyl)aniline (500 mg, 2.42 mmol) and conc. hydrochloric acid aqueous solution (7 mL) was stirred vigorously at 0 °C (ice bath) for 10 min. Subsequently, a solution of sodium nitrite (255 mg, 3.71 mmol, 1.53 equiv.) in water (5 mL) was added in a dropwise manner over a period of 5 min; the mixture was stirred at 0 °C for an additional 30 min. Then, a solution of sodium azide (324 mg, 4.99 mmol, 2.06 equiv.) in water (5 mL) was added dropwise at 0 °C, and the reaction mixture was stirred vigorously at room temperature for a further 3 h. Finally, conc. sodium hydroxide aqueous solution was added to bring the mixture to pH 7, which was then extracted with ethyl acetate (3 × 35 mL). The extracts were combined, washed with distilled water (1 × 50 mL), dried over anhydrous sodium sulfate, filtered and concentrated under reduced pressure to give compound **A** as a yellow liquid (521 mg, 92%). ^{1}H NMR (300 MHz, $CDCl_3$): δ (ppm) = 7.17-7.14 (m, 2H), 6.93-6.89 (m, 2H), 3.71-3.69 (m, 4H), 2.77-2.72 (m, 2H), 2.56-2.46 (m, 6H). ^{13}C NMR {DEPT-135} (75 MHz, $CDCl_3$): δ (ppm) = 137.9 (ArC), 137.0 (ArC), 130.1 (ArCH), 119.0 (ArCH), 67.0 (CH_2), 60.7 (CH_2), 53.7 (CH_2), 32.7 (CH_2). HR-MS (+ESI): found *m/z* 233.1402 ($[M+H]^+$), $[C_{12}H_{17}N_4O]^+$ requires *m/z* 233.1397 (monoisotopic mass).

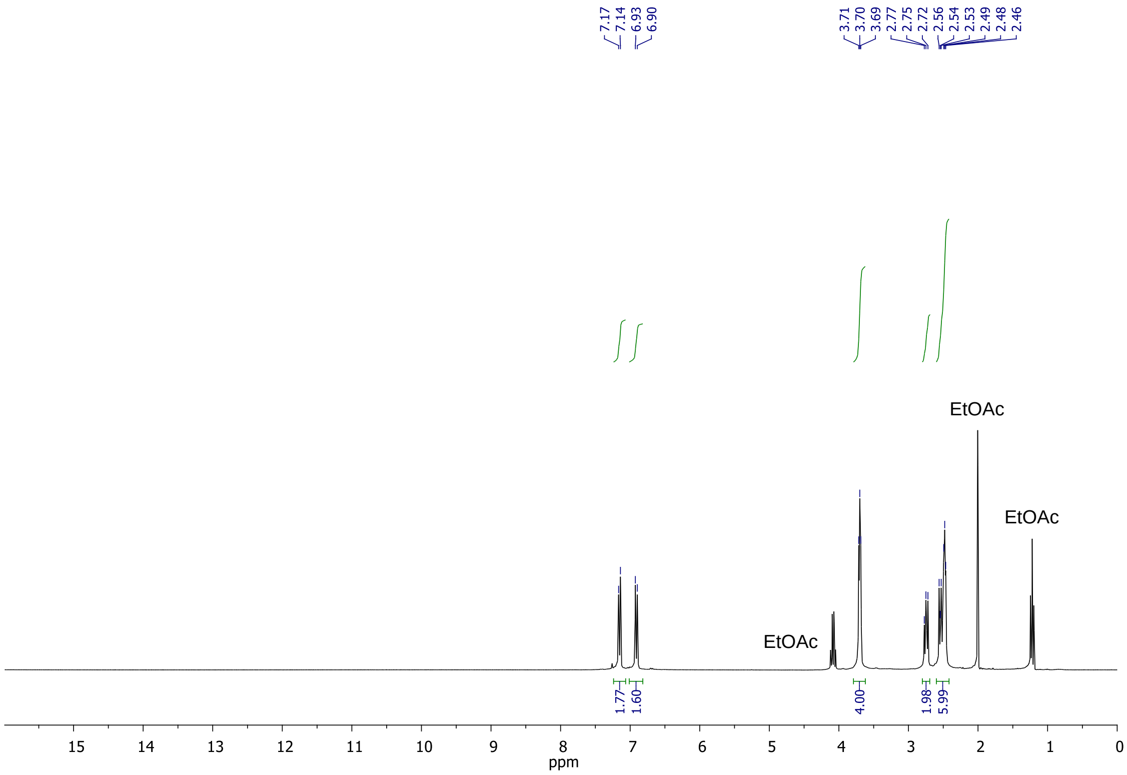

Figure S1. ^{1}H NMR spectrum (300 MHz, $CDCl_3$) for compound **A**.

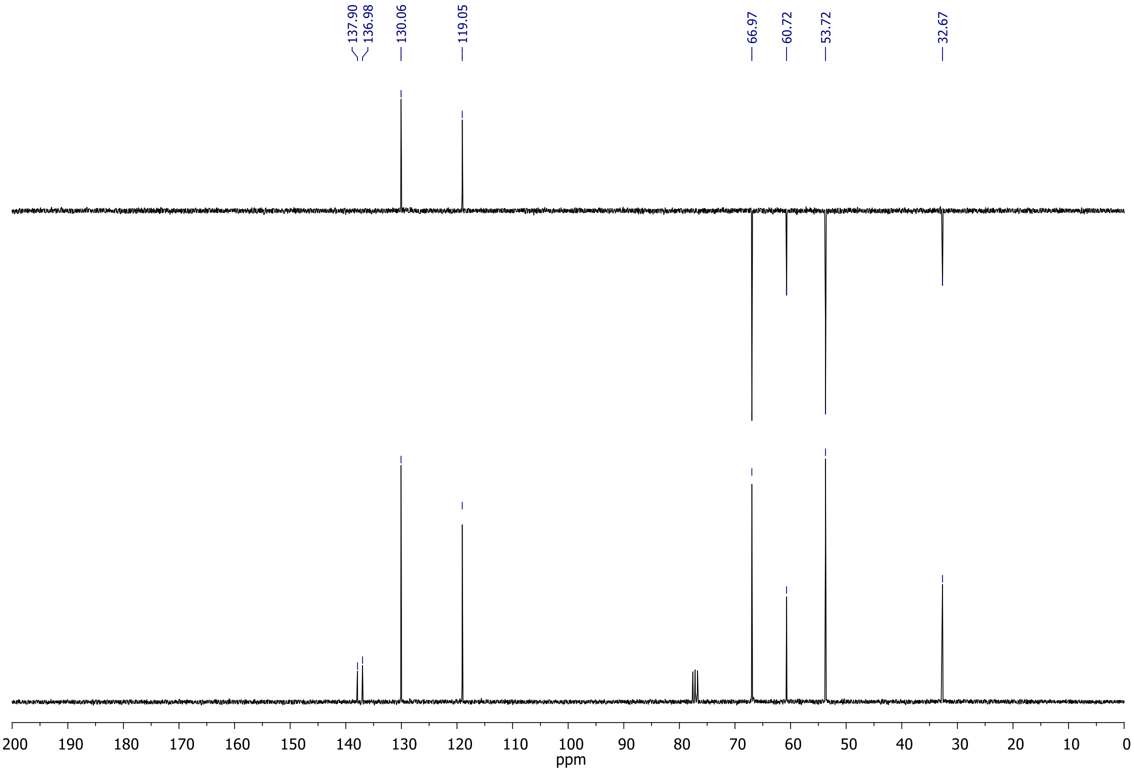

Figure S2. ^{13}C and DEPT-135 NMR spectra (75 MHz, $CDCl_3$) for compound **A**.

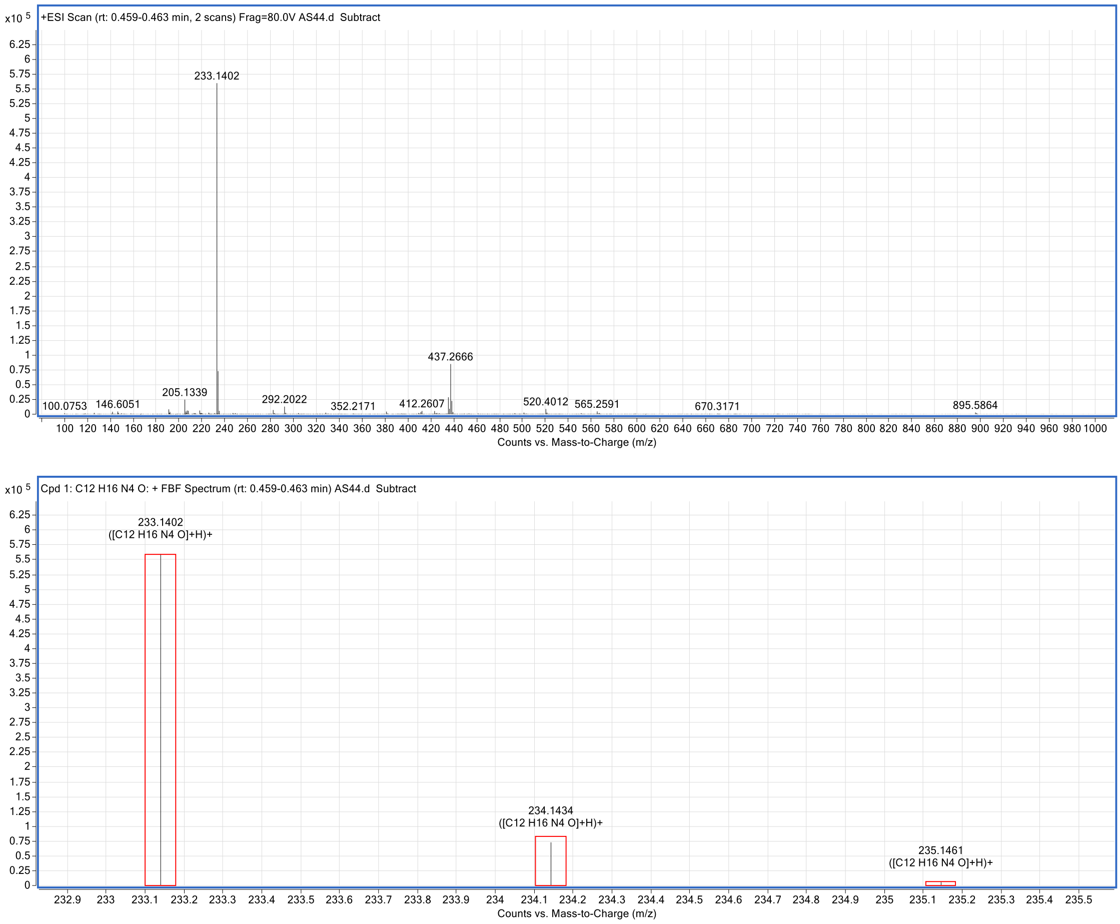

Figure S3. HR-MS (+ESI) spectrum for compound **A**.

1.2.2. Compound B

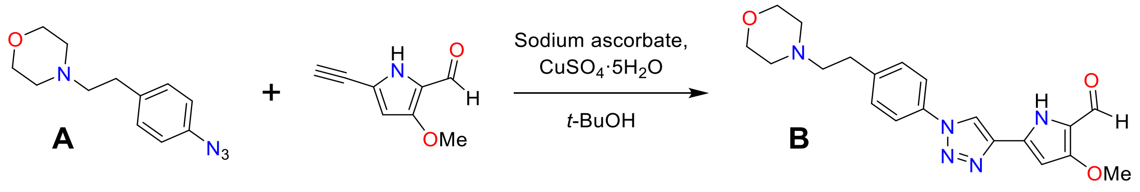

5-ethynyl-3-methoxy-1*H*-pyrrole-2-carbaldehyde[1] (332 mg, 2.22 mmol) and compound **A** (522 mg, 2.25 mmol, 1.01 equiv.) were dissolved partially in *tert*-butanol (10 mL). A solution of (+)-sodium L-ascorbate (88 mg, 0.44 mmol, 0.20 equiv.) in water (5 mL) and a solution of $CuSO_4 \cdot 5H_2O$ (55 mg, 0.22 mmol, 0.10 equiv.) in water (5 mL) were subsequently added to the resulting suspension. The reaction mixture was stirred at room temperature for 18 h and the content of the flask was then poured into water (50 mL); the precipitate was isolated by vacuum filtration, washed with water (3 × 30 mL) and diethyl ether (3 × 50 mL), and dried *in vacuo* to give compound **B** as a brown non-crystalline powder (425 mg, 50%). ^{1}H NMR (300 MHz, DMSO-d_6): δ (ppm) = 12.04 (s, 1H), 9.48 (s, 1H), 9.08 (s, 1H), 7.75 (d, J = 8.1 Hz, 2H), 7.49 (d, J = 8.1 Hz, 2H), 6.52 (s, 1H), 3.89 (s, 3H), 3.64-3.51 (m, 4H), 2.85-2.81 (m, 2H), 2.57 (s_b, 2H), 2.45 (s_b, 2H). ^{13}C NMR {DEPT-135} (75 MHz, DMSO-d_6): δ (ppm) = 173.9 (CHO), 157.7 (ArC), 141.6 (ArC), 140.2 (ArC), 134.4 (ArC), 130.2 (ArCH), 129.4 (ArC), 120.1 (ArCH), 118.7 (ArC), 93.7 (ArCH), 66.1 (CH_2), 59.5 (CH_2), 58.0 (CH_3), 53.2 (CH_2), 31.7 (CH_2). HR-MS (+ESI): found *m/z* 382.1889 ($[M+H]^+$), $[C_{20}H_{24}N_5O_3]^+$ requires *m/z* 382.1874 (monoisotopic mass).

1 Hernando, E.; Capurro, V.; Cossu, C.; Fiore, M.; García-Valverde, M.; Soto-Cerrato, V.; Pérez-Tomás, R.; Moran, O.; Zegarra-Moran, O.; Quesada, R. Small molecule anionophores promote transmembrane anion permeation matching CFTR activity. *Sci. Rep.*, **2018**, *8*, 2608, doi: 10.1038/s41598-018-20708-3.

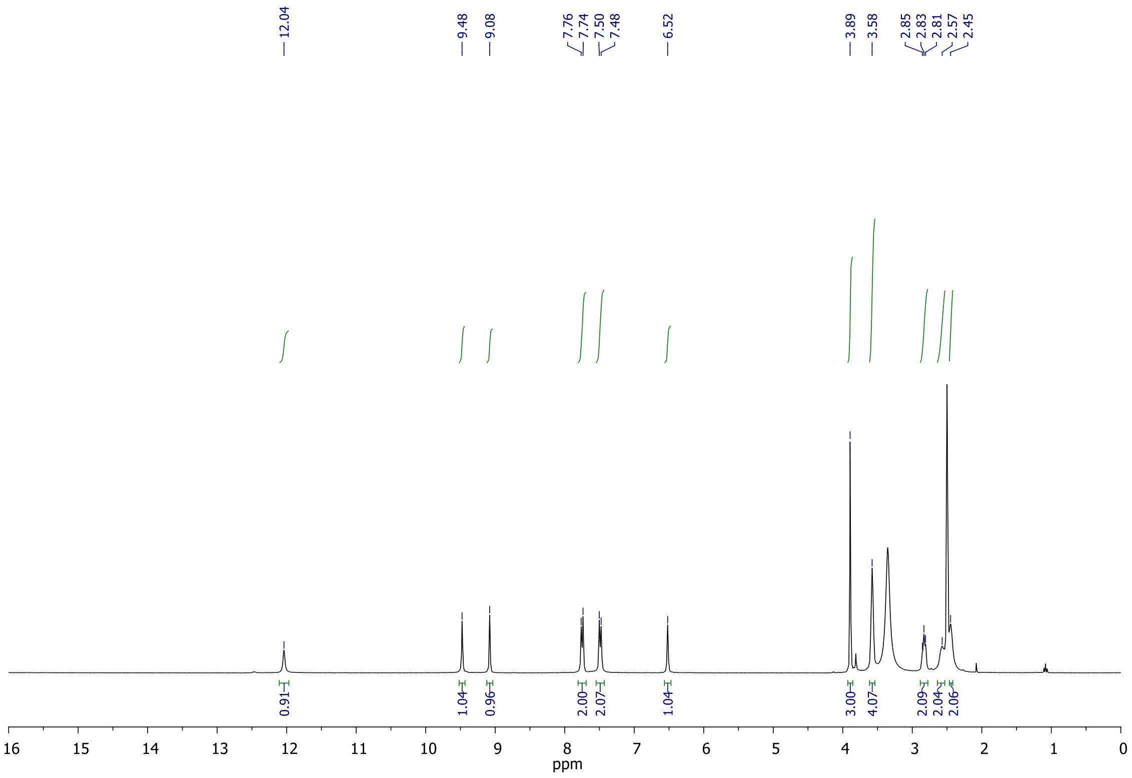

Figure S4. ^{1}H NMR spectrum (300 MHz, DMSO-d_6) for compound **B**.

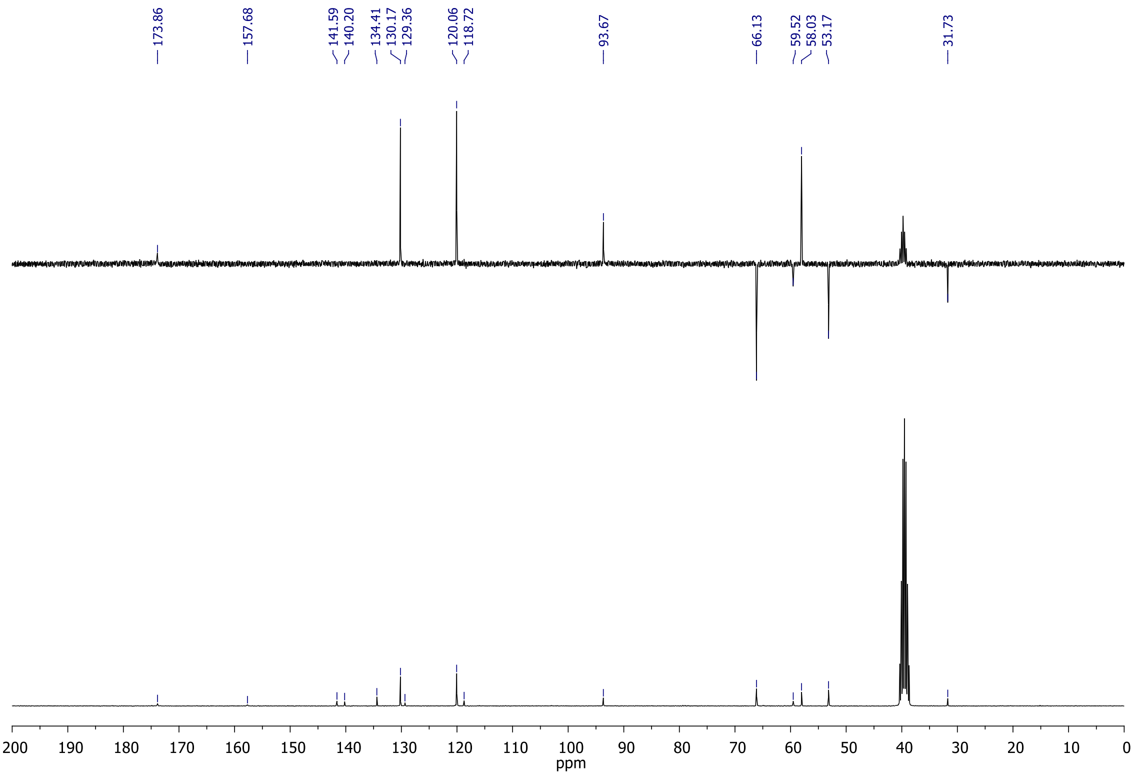

Figure S5. ^{13}C and DEPT-135 NMR spectra (75 MHz, DMSO-d_6) for compound **B**.

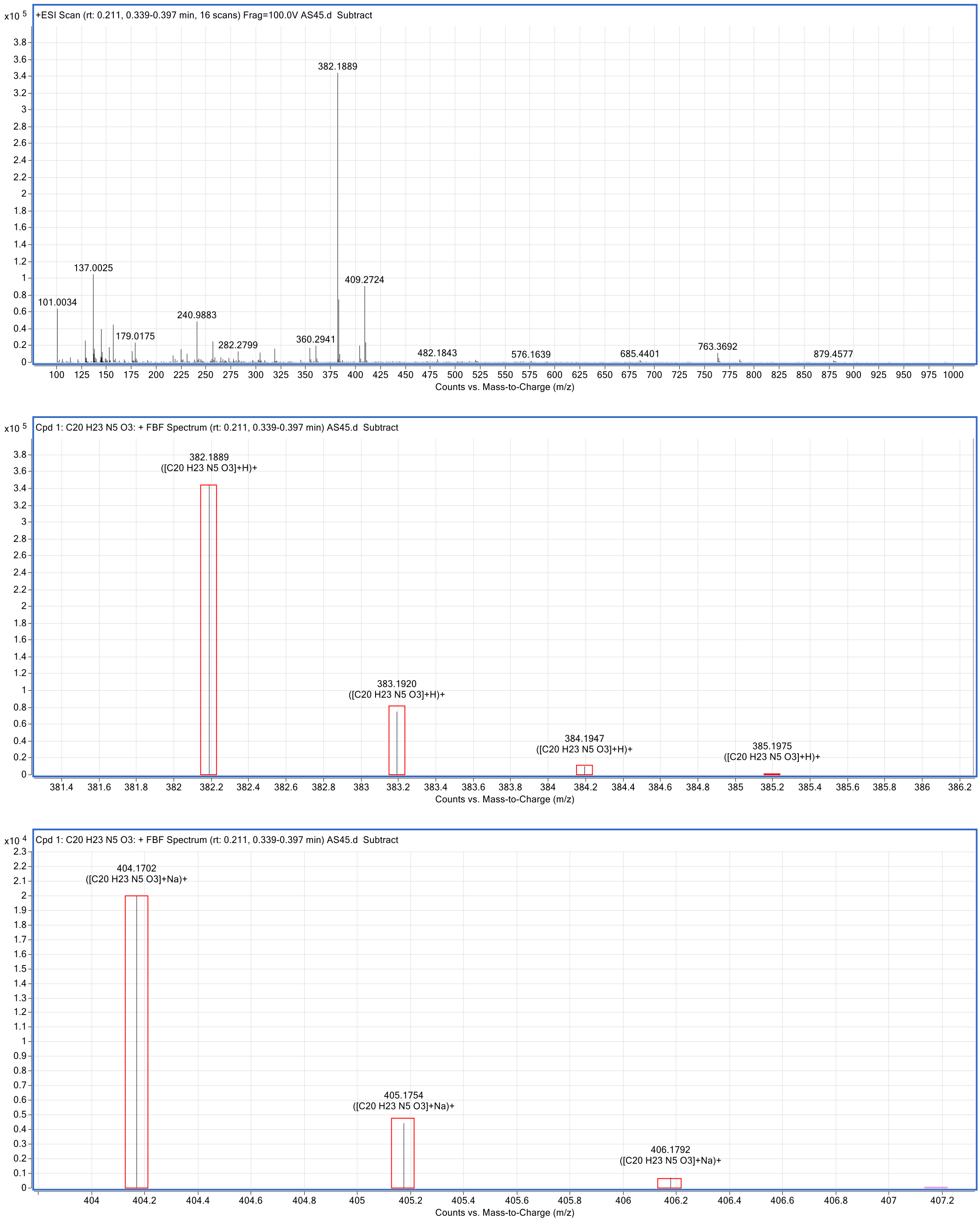

Figure S6. HR-MS (+ESI) spectrum for compound **B**.

1.3. Click-tambjamine

1.3.1. Compound LAI-1

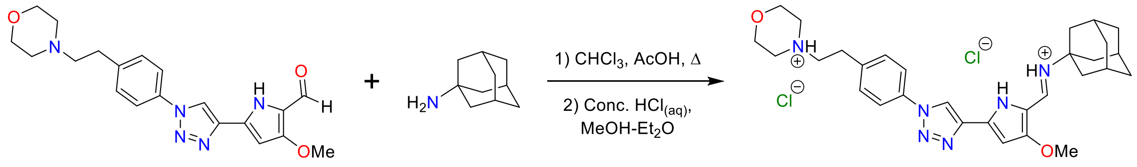

B **LAI-1**

A mixture of aldehyde **B** (102 mg, 0.27 mmol), 1-adamantylamine (44 mg, 0.29 mmol, 1.10 equiv.) and glacial acetic acid (100 µL) in chloroform (15 mL) was stirred at 65 °C for 16 h. Upon cooling to room temperature, the chloroform was evaporated under reduced pressure and the residue redissolved in dichloromethane (15 mL). The solution of the crude compound was washed with distilled water (3 × 15 mL), dried over anhydrous sodium sulfate, filtered and concentrated to dryness. The product was dissolved in a mixture of methanol (3 mL) and diethyl ether (10 mL); conc. hydrochloric acid aqueous solution (120 µL) was added and the mixture was stirred at room temperature for 16 h. The precipitate was isolated by vacuum filtration, washed with diethyl ether (5 mL) and dried *in vacuo* to give compound **LAI-1** as a dark brown non-crystalline solid (99 mg, 63%). ^{1}H NMR (300 MHz, DMSO-d_6): δ (ppm) = 13.35 (s, 1H), 11.32 (s, 1H), 11.27 (s, 1H), 9.35 (s, 1H), 8.04 (d, J = 15.6 Hz, 1H), 7.90 (d, J = 8.2 Hz, 2H), 7.58 (d, J = 8.2 Hz, 2H), 6.68 (s, 1H), 4.00-3.98 (m, 5H), 3.82 (t, J = 12.0 Hz, 2H), 3.50 (d, J = 12.5 Hz, 2H), 3.22-3.05 (m, 6H), 2.16 (s, 3H), 1.98 (s, 6H), 1.67 (s, 6H). ^{13}C NMR {DEPT-135} (75 MHz, DMSO-d_6): δ (ppm) = 162.4 (ArC), 141.2 (ArCH), 139.1 (ArC), 138.5 (ArC), 136.4 (ArC), 134.8 (ArC), 130.3 (ArCH), 122.1 (ArCH), 120.7 (ArCH), 110.6 (ArC), 94.2 (ArCH), 63.1 (CH_2), 58.9 (CH_3), 56.8 (C_q), 56.1 (CH_2), 50.9 (CH_2), 40.9 (CH_2), 35.0 (CH_2), 28.6 (CH), 28.4 (CH_2). HR-MS (+ESI): found m/z 258.1609 ($[M+2H]^{2+}$), $[C_{30}H_{40}N_6O_2]^{2+}$ requires m/z 258.1601 (diisotopic mass).

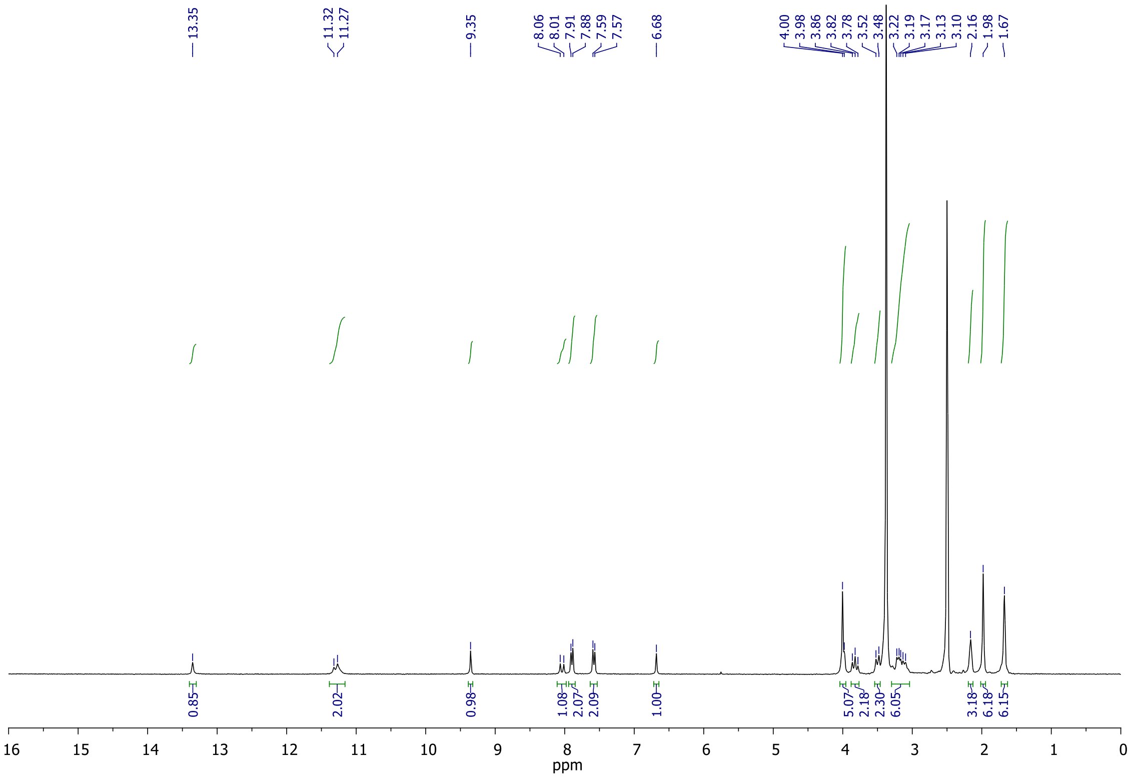

Figure S7. ^{1}H NMR spectrum (300 MHz, DMSO-d_6) for compound **LAI-1**.

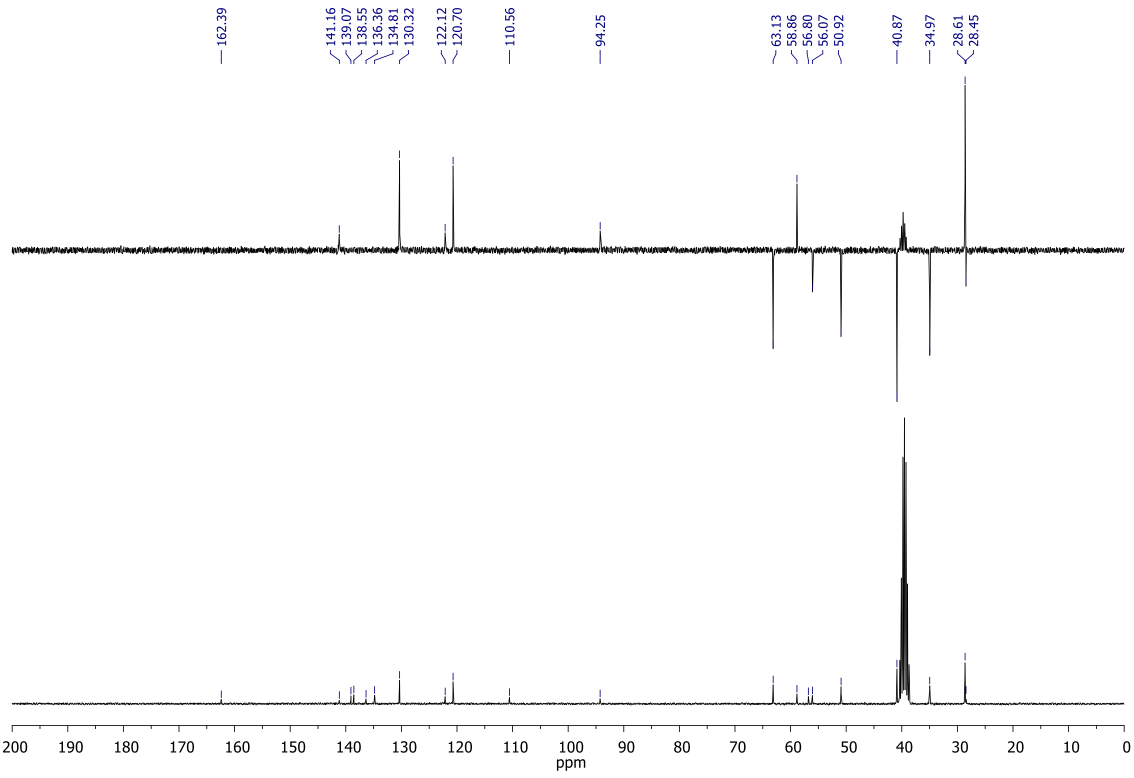

Figure S8. ^{13}C and DEPT-135 NMR spectra (75 MHz, DMSO-d_6) for compound **LAI-1**.

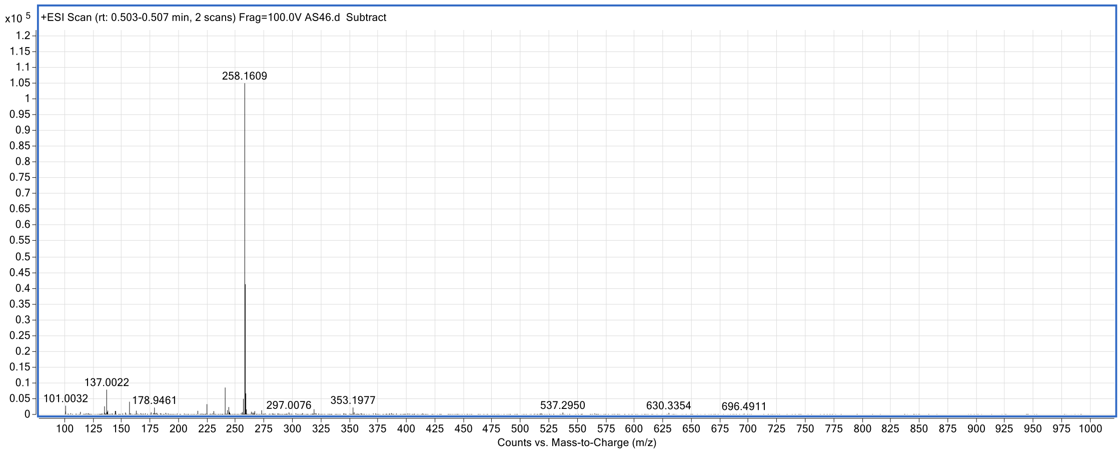

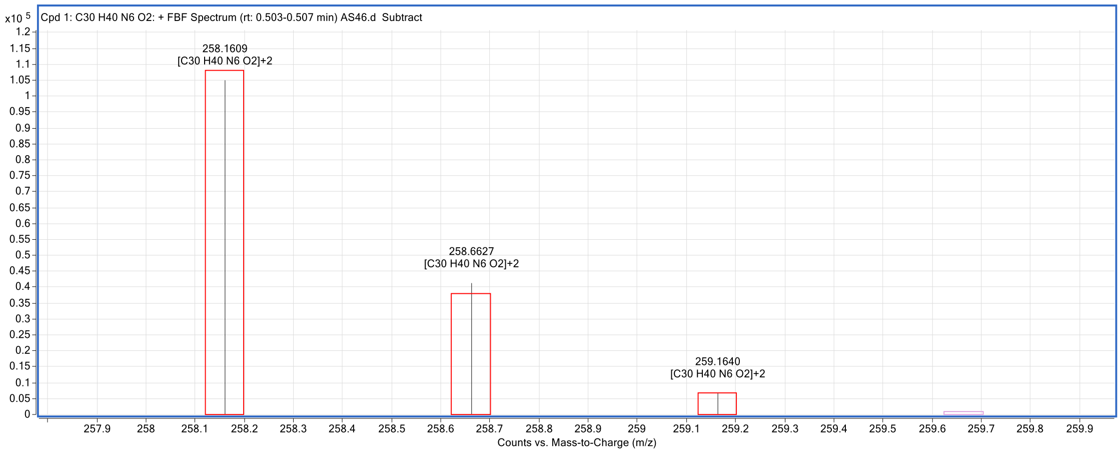

Figure S9. HR-MS (+ESI) spectrum for compound **LAI-1**.

2. ABSORPTION AND EMISSION SPECTRA OF COMPOUND LAI-1

2.1. Absorption spectrum in DMSO

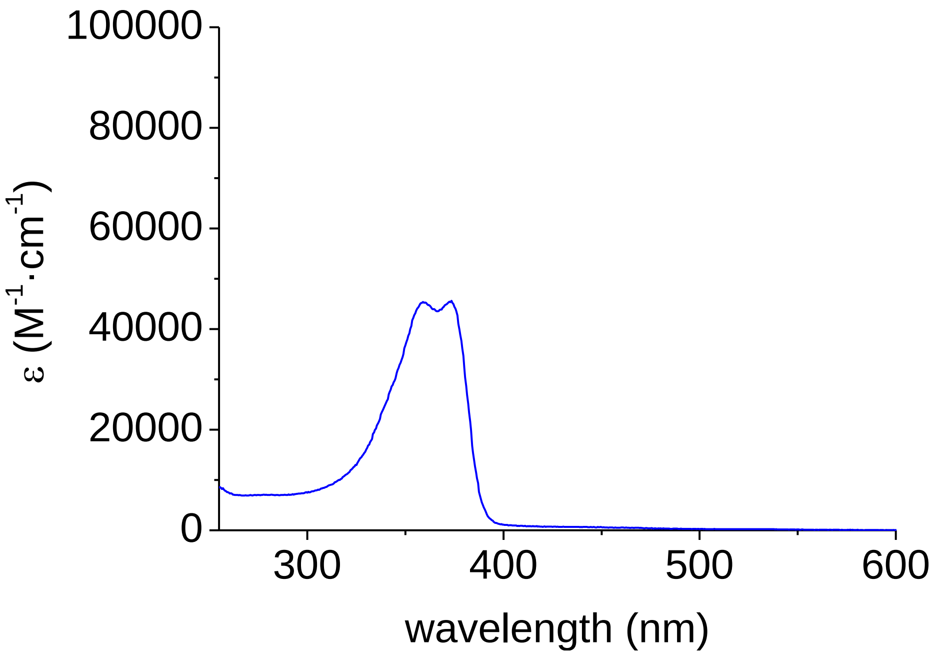

Figure S10. Absorption spectrum recorded for a $2{\cdot}10^{-5}$ M solution of compound **LAI-1** in DMSO.

2.2. Emission spectra in DMSO

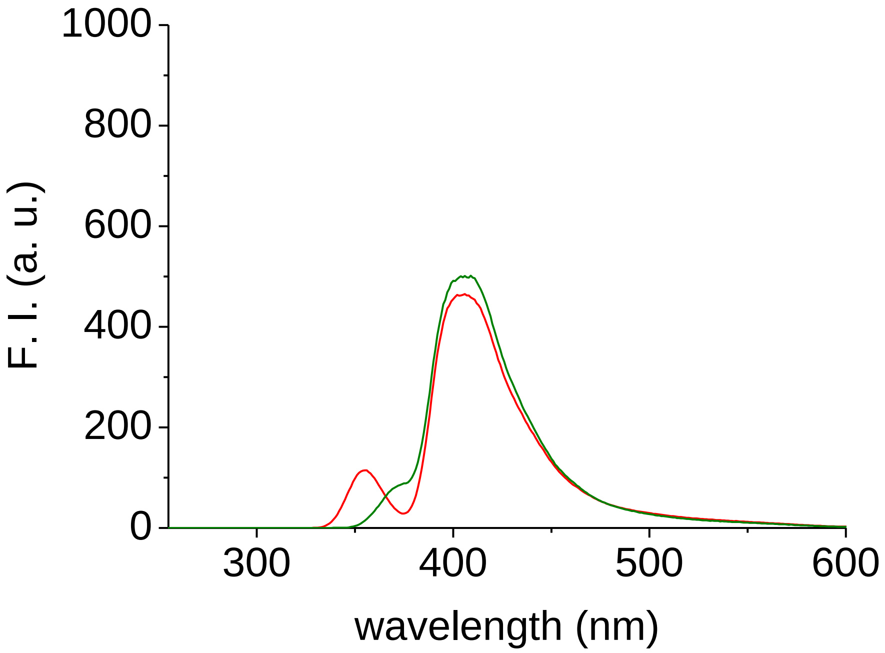

Figure S11. Emission spectra recorded for a $2{\cdot}10^{-5}$ M solution of compound **LAI-1** in DMSO. The sample was excited at 359 (red line) and 373 nm (green line), the wavelengths of the maxima of its absorption spectrum. In both cases, EX Slit = 20.0 nm and EM Slit = 10.0 nm.

2.3. Emission spectra inside cells

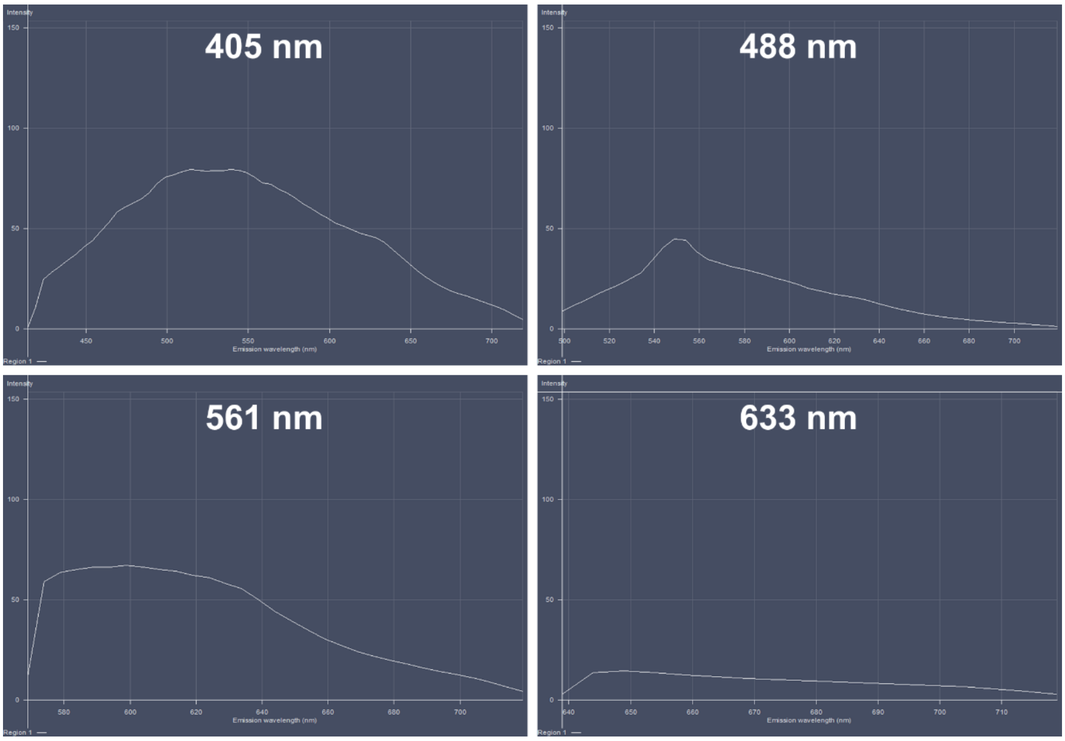

Figure S12: Emission spectra recorded in A549 cells after 3 h of treatment with 10^{-5} M of compound **LAI-1**. Cells were excited using different lasers (405, 488, 561 and 633 nm lasers) and emission wavelength scan (EM slit: 10 nm) was collected for each laser. The highest fluorescence emissions were obtained at around 540 nm after 405 nm excitation and around 600 nm after 561 nm laser excitation. Images were obtained from Carl Zeiss LSM 880 spectral confocal laser scanning microscope (Carl Zeiss Microscopy GmbH, Jena, Germany) and data were processed with ZEN 2 Blue edition software (Zeiss).

3. TRANSMEMBRANE ANION TRANSPORT EXPERIMENTS IN VESICLES

3.1. Preparation of phospholipid vesicles

A chloroform solution of 1-palmitoyl-2-oleoyl-*sn*-glycero-3-phosphocoline (POPC) (20 mg/mL) (Sigma Aldrich) (or a 7:3 POPC:cholesterol mixture, in the case of HPTS-based assays) was evaporated *in vacuo* using a rotary evaporator and the resulting film was dried under high vacuum for, at least, 2 h. Different aqueous solutions were used to rehydrate the lipid film: (a) ISE assays: 489 mM NaCl, 5 mM NaH_2PO_4, I.S. 500 mM, pH 7.2, for Cl^-/NO_3^- exchange experiments, or 451 mM NaCl, 20 mM NaH_2PO_4, I.S. 500 mM, pH 7.2, for Cl^-/HCO_3^- exchange experiments; (b) Carboxyfluorescein-based assays: 451 mM NaCl, 20 mM NaH_2PO_4, 50 mM CF, I.S. 500 mM, pH 7.2; (c) HPTS-based assays: 126.2 mM $NaNO_3$, 10 mM NaH_2PO_4, 1 mM HPTS, pH 6.2. The resulting suspension was vortexed and subjected to nine freeze-thaw cycles; subsequently, it was extruded twenty-nine times through a polycarbonate membrane (200 nm) employing a LiposoFast basic extruder (Avestin, Inc.). The resulting unilamellar vesicles were: (a) ISE assays: dialyzed against a $NaNO_3$ aqueous solution (489 mM $NaNO_3$, 5 mM NaH_2PO_4, I.S. 500 mM, pH 7.2, for Cl^-/NO_3^- exchange experiments) or a Na_2SO_4 aqueous solution (150 mM Na_2SO_4, 20 mM NaH_2PO_4, I.S. 500 mM, pH 7.2, for Cl^-/HCO_3^- exchange experiments) to remove the unencapsulated chloride; (b) Carboxyfluorescein-based assays: subjected to size-exclusion chromatography, using Sephadex G-25 as the stationary phase and a Na_2SO_4 aqueous solution (150 mM Na_2SO_4, 20 mM NaH_2PO_4, I.S. 500 mM, pH 7.2) as the mobile phase, to remove the unencapsulated carboxyfluorescein; (c) HPTS-based assays: subjected to size-exclusion chromatography, using Sephadex G-25 as the stationary phase and the inner solution without HPTS (126.2 mM $NaNO_3$, 10 mM NaH_2PO_4, pH 6.2) as the mobile phase, to remove the unencapsulated HPTS. Vesicles were collected in a 10-mL volumetric flask, using either the external solution (ISE and carboxyfluorescein-based assays) or the inner one without the probe (HPTS-based assays) to bring the suspension to the desired volume.

3.2. ISE transport experiments

Unilamellar vesicles (mean diameter: 200 nm) made of POPC and containing a NaCl aqueous solution (489 mM NaCl, 5 mM NaH_2PO_4, I.S. 500 mM, pH 7.2, for Cl^-/NO_3^- exchange experiments, or 451 mM NaCl, 20 mM NaH_2PO_4, I.S. 500 mM, pH 7.2, for Cl^-/HCO_3^- exchange experiments) were dispersed in a $NaNO_3$ aqueous solution (489 mM $NaNO_3$, 5 mM NaH_2PO_4, I.S. 500 mM, pH 7.2, for Cl^-/NO_3^- exchange experiments) or a Na_2SO_4 aqueous solution (150 mM Na_2SO_4, 20 mM NaH_2PO_4, I.S. 500 mM, pH 7.2, for Cl^-/HCO_3^- exchange experiments), the final

lipid concentration during the assays being 0.5 mM and the final volume 5 mL. A certain volume of a solution of compound **LAI-1** in DMSO (or the blank, DMSO, 12.5 µL) was added at t = 0 s, and the chloride released was monitored for 300 s with a chloride-selective electrode (HACH 9652C). At t = 300 s a surfactant (Triton-X, 20% dispersion in water, 20 µL) was added to lyse the vesicles and free all the encapsulated chloride. This value was considered as 100 % chloride release and employed as such. Regarding the Cl^-/HCO_3^- exchange assays, a 500 mM $NaHCO_3$ aqueous solution prepared with the Na_2SO_4 one (150 mM Na_2SO_4, 20 mM NaH_2PO_4, I.S. 500 mM, pH 7.2) was added at t = -10 s to the vesicles suspension, the HCO_3^- concentration during the assay being 40 mM. The rest of the experimental procedure is similar to that described previously.

Table S1. Transport activities (Cl^-/NO_3^- and Cl^-/HCO_3^- exchanges) expressed as EC_{50} (nM) and Hill parameter (*n*) for compound **LAI-1**.

**Cl^-/NO_3^- exchange**		**Cl^-/HCO_3^- exchange**	
**EC_{50}**	***n***	**EC_{50}**	***n***
152 ± 12	1.1 ± 0.1	522 ± 65	1.0 ± 0.1

Cl^-/NO_3^- exchange assays

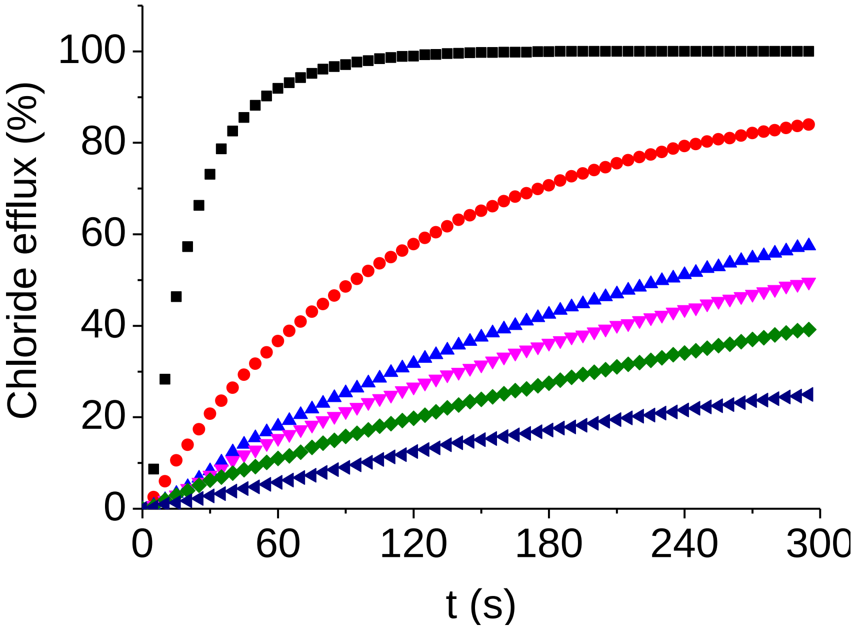

Figure S13. Chloride efflux promoted by compound **LAI-1** (5 μM, black; 0.5 μM, red; 0.25 μM, light blue; 0.15 μM, pink; 0.1 μM, green; 0.05 μM, dark blue) in unilamellar POPC vesicles. Vesicles were loaded with a 489 mM NaCl solution buffered at pH 7.2 with 5 mM NaH_2PO_4 and dispersed in a 489 mM $NaNO_3$ solution buffered at pH 7.2. Each trace represents the average of at least three trials, performed with at least three batches of vesicles.

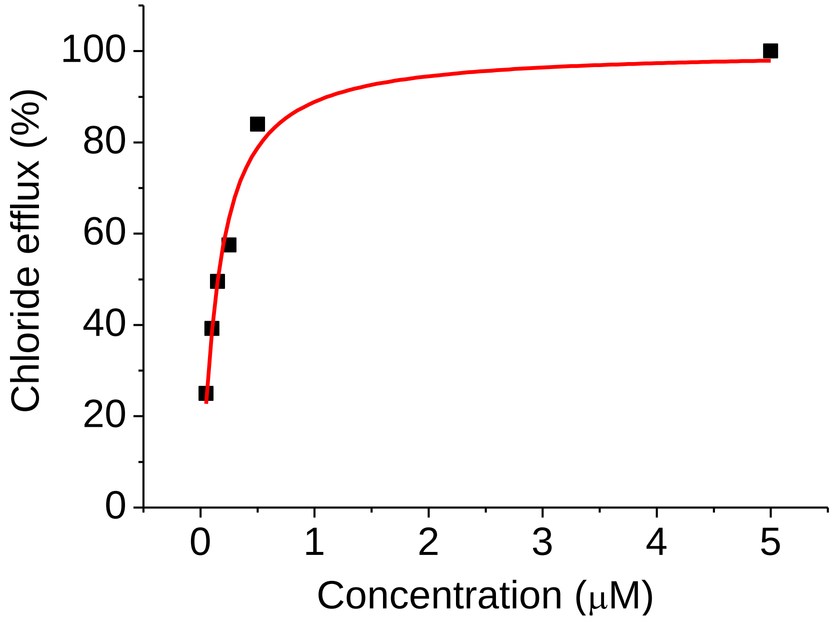

Figure S14. Hill analysis for compound **LAI-1**.

Cl⁻/HCO$_3$⁻ exchange assays

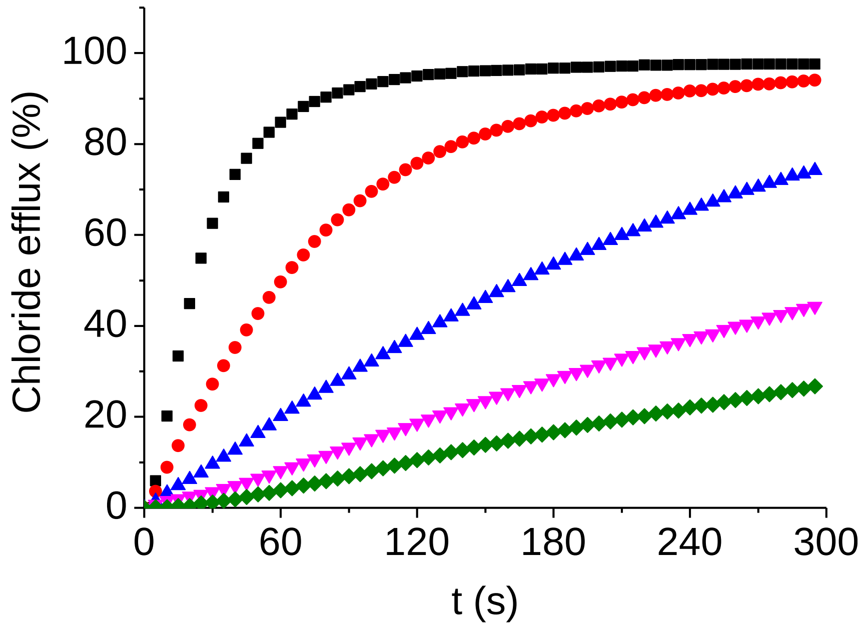

Figure S15. Chloride efflux promoted by compound **LAI-1** (15 µM, black; 5 µM, red; 1.5 µM, blue; 0.5 µM, pink; 0.15 µM, green) in unilamellar POPC vesicles. Vesicles, loaded with a NaCl solution (451 mM NaCl and 20 mM NaH_2PO_4, pH 7.2), were immersed in a Na_2SO_4 solution (150 mM Na_2SO_4, 40 mM $NaHCO_3$ and 20 mM NaH_2PO_4, pH 7.2). Each trace represents the average of at least three trials, performed with at least three batches of vesicles.

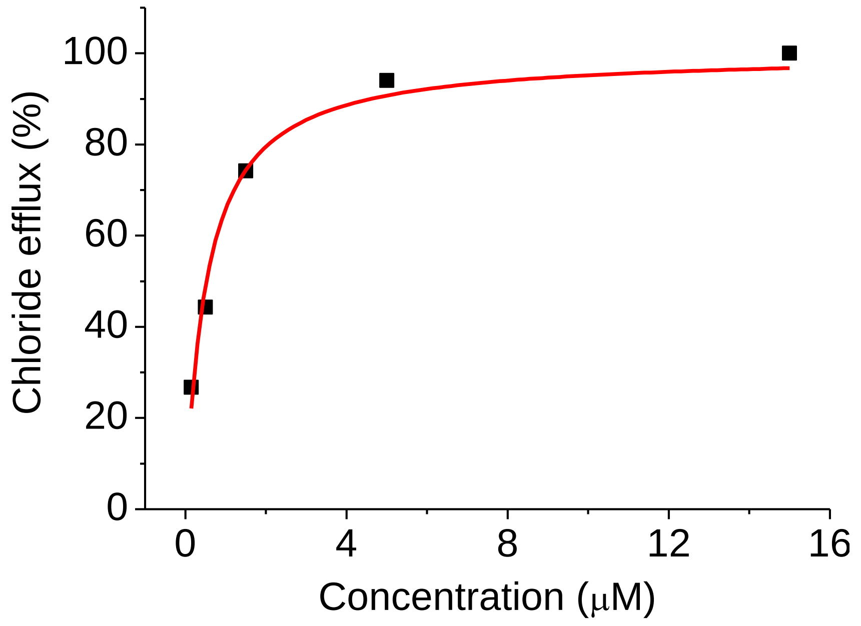

Figure S16. Hill analysis for compound **LAI-1**.

3.3. Emission spectroscopy transport experiments

3.3.1. Carboxyfluorescein-based assays

Vesicles made of POPC were loaded with a NaCl aqueous solution (451 mM NaCl, 20 mM NaH_2PO_4, 50 mM CF, I.S. 500 mM, pH 7.2) and treated according to the procedure described in *Section 3.1*. The experiments were performed in 1-cm disposable cells, the final POPC concentration in the cuvette being 0.05 mM and the total volume 2.5 mL. At t = 60 s an aliquot of a solution of compound **LAI-1** in DMSO (or the blank, DMSO, 1.25 µL) was added, and emission changes were recorded for 300 s. At t = 360 s a pulse of a detergent (Triton-X, 20% dispersion in water, 20 µL) was added to lyse the vesicles and free all the entrapped CF. The obtained emission value was regarded as 100% release and used to normalize the data.

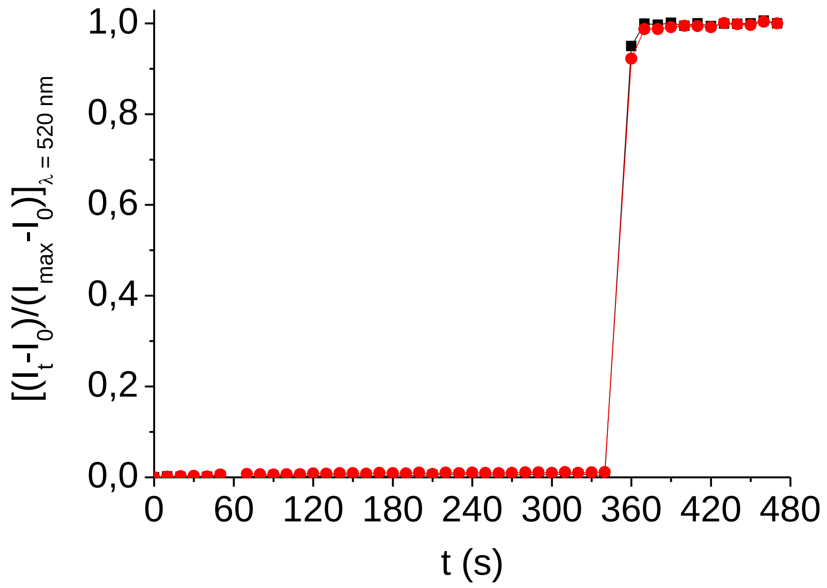

Figure S17. Carboxyfluorescein leakage observed upon addition of compound **LAI-1** to POPC vesicles (0.05 mM). Vesicles (loaded with a 451 mM NaCl solution buffered at pH 7.2 with 20 mM NaH_2PO_4, and containing 50 mM CF; I.S. 500 mM) were suspended in a Na_2SO_4 solution (150 mM Na_2SO_4, buffered at pH 7.2 with 20 mM NaH_2PO_4; I.S. 500 mM). At t = 60 s compound **LAI-1** was added (red trace; 0.5 µM, 1% mol carrier to lipid), while at t = 360 s the detergent (20 µL) was added. The blank is DMSO (black trace; 1.25 µL). Each trace represents the average of three trials, performed with three batches of vesicles.

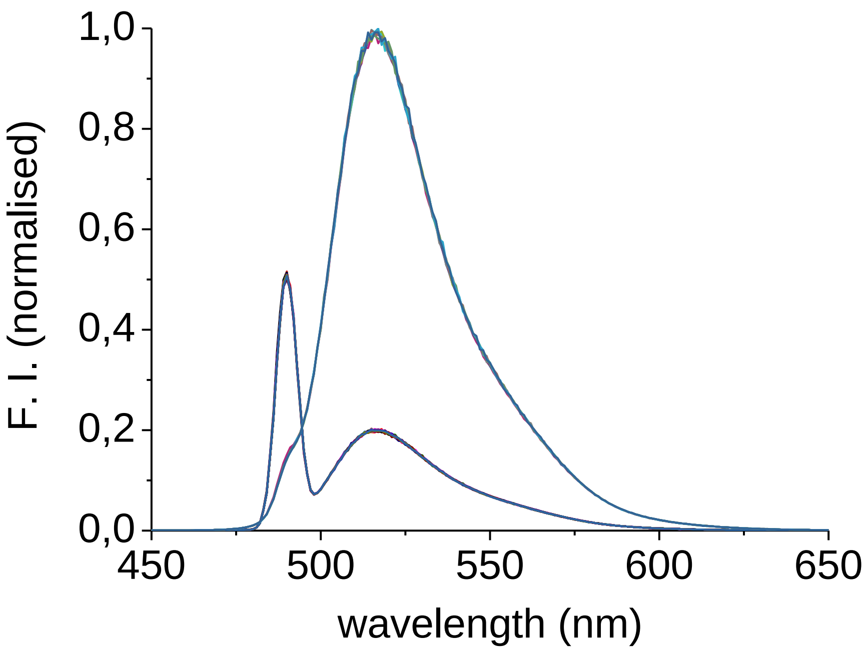

Figure S18. Carboxyfluorescein normalized fluorescence intensity recorded upon addition of compound **LAI-1** to POPC vesicles (0.05 mM). Vesicles (loaded with a 451 mM NaCl solution buffered at pH 7.2 with 20 mM NaH_2PO_4, and containing 50 mM CF; I.S. 500 mM) were suspended in a Na_2SO_4 solution (150 mM Na_2SO_4, buffered at pH 7.2 with 20 mM NaH_2PO_4; I.S. 500 mM). At t = 60 s compound **LAI-1** was added (0.5 µM, 1% mol carrier to lipid), while at t = 360 s the detergent (20 µL) was added. Each trace represents the average of three trials, performed with three batches of vesicles.

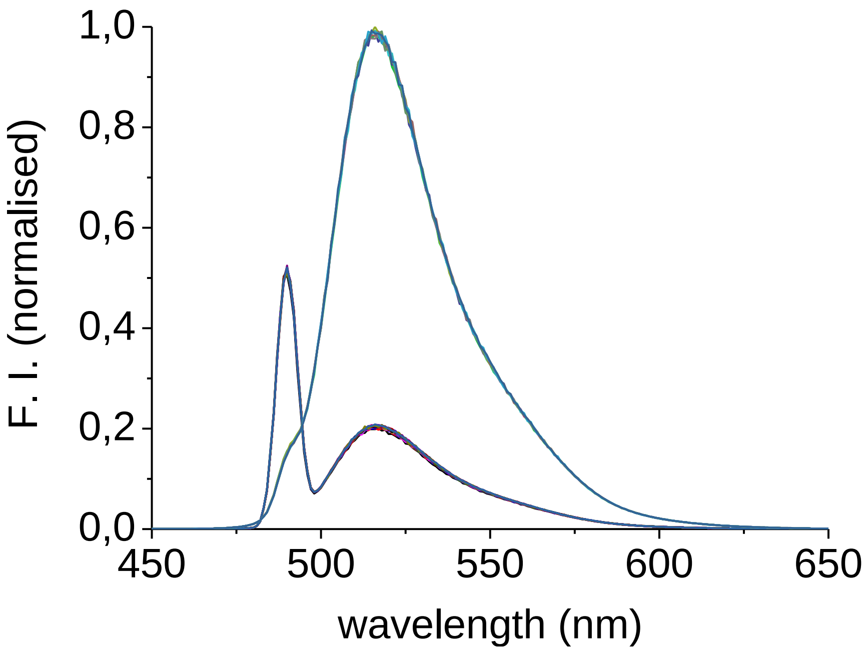

Figure S19. Carboxyfluorescein normalized fluorescence intensity recorded upon addition of **DMSO** to POPC vesicles (0.05 mM). Vesicles (loaded with a 451 mM NaCl solution buffered at pH 7.2 with 20 mM NaH_2PO_4, and containing 50 mM CF; I.S. 500 mM) were suspended in a Na_2SO_4 solution (150 mM Na_2SO_4, buffered at pH 7.2 with 20 mM NaH_2PO_4; I.S. 500 mM). At t = 60 s **DMSO** was added (1.25 µL), while at t = 360 s the detergent (20 µL) was added. Each trace represents the average of three trials, performed with three batches of vesicles.

3.3.2. HPTS-based assays

First of all, a calibration curve matching I_{460}/I_{403}, the relationship between the emission intensities collected at 510 nm when exciting the sample at 460 nm and 403 nm (the excitation wavelengths of the dye's deprotonated and protonated forms, respectively) of an HPTS aqueous solution (15 nM), prepared with a $NaNO_3$ aqueous solution (126.2 mM $NaNO_3$, 10 mM NaH_2PO_4), and the pH was built. In order to do it, aliquots of a NaOH aqueous solution (0.5 M), prepared with a $NaNO_3$ aqueous solution (126.2 mM $NaNO_3$, 10 mM NaH_2PO_4), were successively added to the HPTS solution, and after each addition I_{460}/I_{403} and the pH value of the solution were recorded. Data were fitted to an *S*-logistic model, which provided an R^2 = 0.9999.

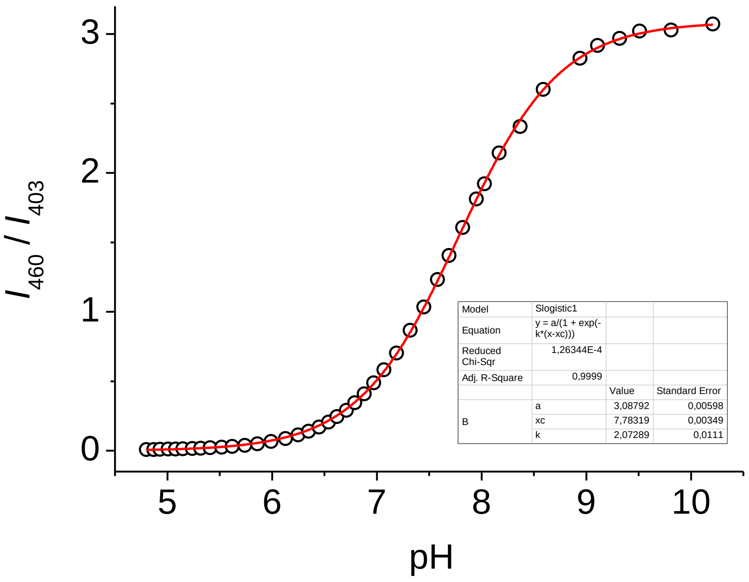

Figure S20. Calibration curve obtained for HPTS (126.2 mM $NaNO_3$, 10 mM NaH_2PO_4, 15 nM HPTS). Data were fitted to an *S*-logistic model (Origin Pro®).

7:3 POPC:cholesterol vesicles were loaded with a $NaNO_3$ aqueous solution (126.2 mM $NaNO_3$, 10 mM NaH_2PO_4, 1 mM HPTS, pH 6.2) and treated according to the procedure described in *Section 3.1*. The experiments were performed in 1-cm disposable cells, the final POPC concentration in the cuvette being 0.5 mM and the final volume 2.5 mL. Just before starting the measurements the required volume of the vesicles stock solution was suspended in the outer solution (126.2 mM $NaNO_3$, 10 mM NaH_2PO_4, pH 7.5). At t = 60 s an aliquot of a solution of compound **LAI-1** in DMSO (or the blank, DMSO, 6.25 µL) was added, and the ratio of emission intensities recorded at 510 nm by excitation of the sample at 460 and 403 nm was recorded for five more minutes. At t = 360 s a detergent (Triton-X, 20% dispersion in water, 20 µL) was added, to lyse the vesicles and balance the pH.

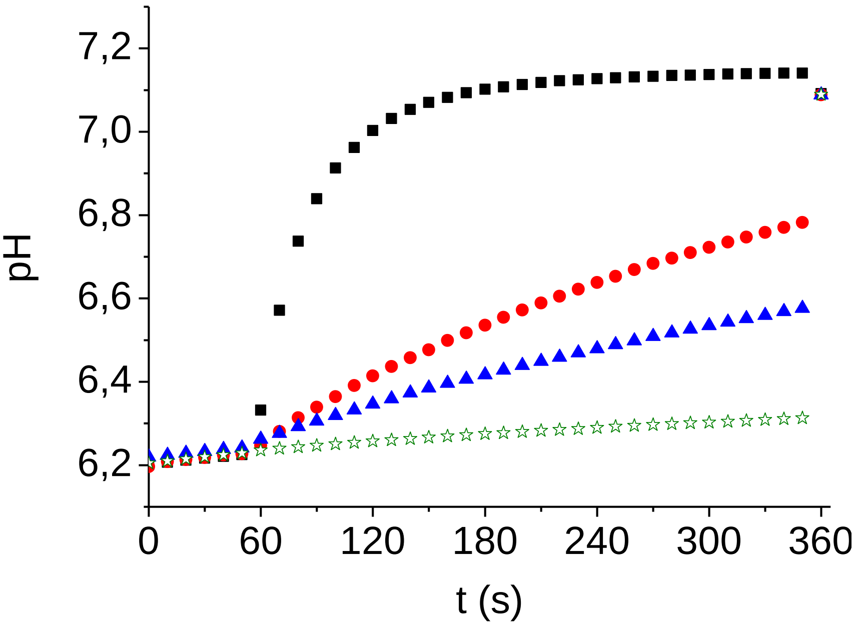

Figure S21. Variation of pH upon addition of compound **LAI-1** (50 nM, black; 5 nM, red; 2.5 nM, blue; blank, green) to 7:3 POPC:cholesterol vesicles (0.5 mM POPC). Vesicles, loaded with a $NaNO_3$ buffered aqueous solution (126.2 mM $NaNO_3$, 10 mM NaH_2PO_4, 1 mM HPTS, pH 6.2), were suspended in a $NaNO_3$ buffered aqueous solution (126.2 mM $NaNO_3$, 10 mM NaH_2PO_4, pH 7.5) just before starting the measurements. At t = 60 s the compound (or the blank, DMSO, 6.25 µL) was added, and at t = 360 s a detergent (Triton-X, 20% dispersion in water, 20 µL) was added. Each trace corresponds to the average of at least three trials performed with three batches of vesicles. The pH value obtained after mixing the inner and outer solutions in the same proportions used during the assays was 7.20 (measured with a pH-meter).

4. SUPPLEMEMENTARY BIOLOGICAL RESULTS

4.1. Cell viability studies

Table S2. Inhibitory concentration (IC); IC_{25}, IC_{50} and IC_{75} values after chloroquine (CQ) treatment for A549, SW900 and DMS53 cell lines. Data show mean value ± SD.

CQ (µM)	A549	SW900	DMS53
**IC_{25}**	140.37 ± 36.83	137.11 ± 13.90	75.58 ± 17.64
**IC_{50}**	213.48 ± 28.98	263.05 ± 45.65	156.11 ± 27.34
**IC_{75}**	329.92 ± 31.36	508.78 ± 141.84	324.65 ± 48.87

Table S3. Inhibitory concentration (IC); IC_{25}, IC_{50} and IC_{75} values after 3-methyladenine (3-MA) treatment for A549, SW900 and DMS53 cell lines. Data show mean value ± SD.

3-MA (µM)	A549	SW900	DMS53
**IC_{25}**	>10000	>10000	>10000
**IC_{50}**	>10000	>10000	>10000
**IC_{75}**	>10000	3417.16 ± 505.57	>10000

4.2. Blockage of autophagosomes and lysosomes fusion

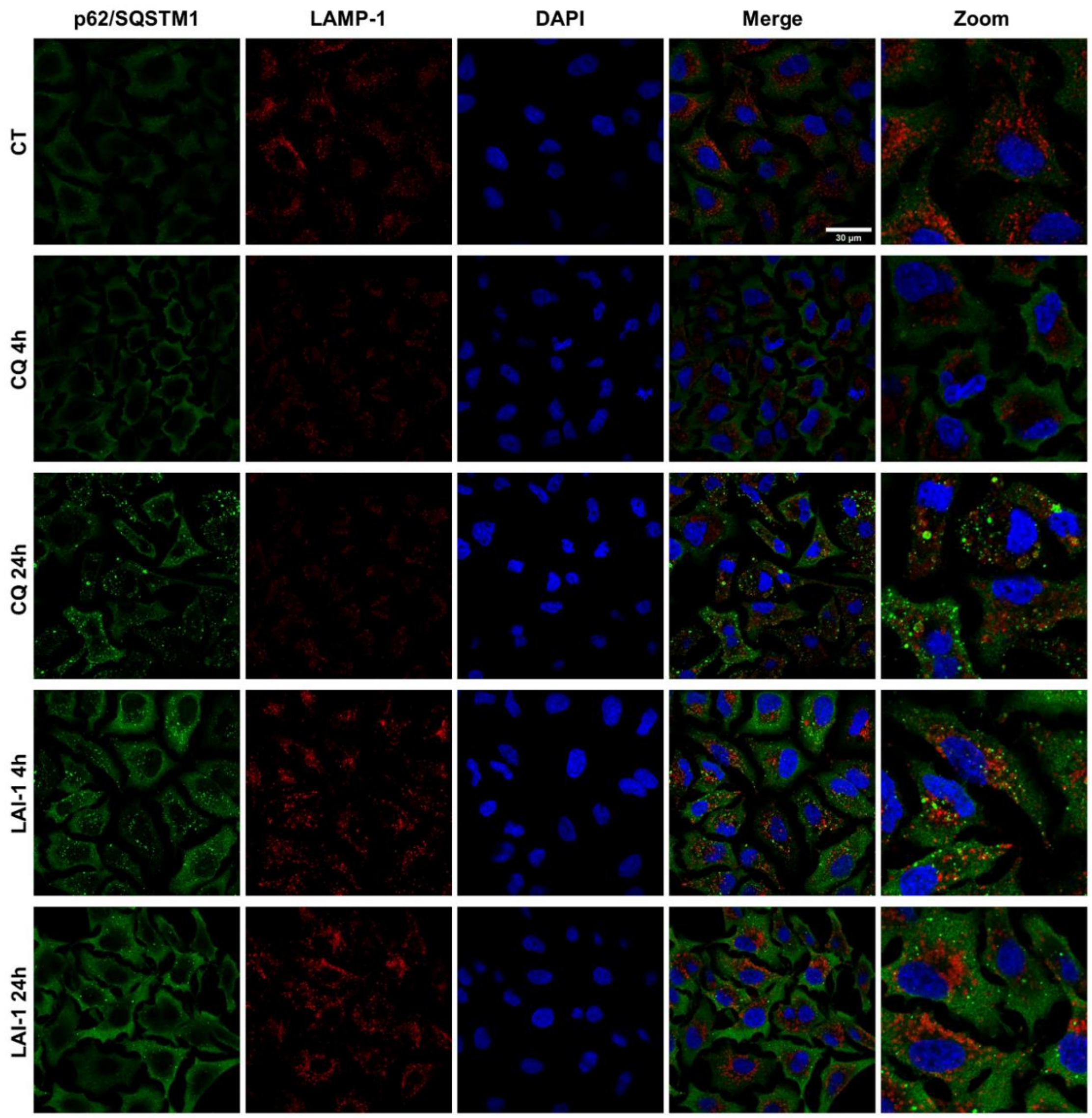

Figure S22. Subcellular localization of p62/SQSTM1 and LAMP-1. A549 cells were seeded for 24 h on coverslips and then treated with **LAI-1** (15 µM) and chloroquine (CQ, 50 µM) for different times. The fusion between autophagosomes, marked with p62/SQSTM1 (green), and lysosomes, marked with LAMP-1 (red), was studied in merged images. Zoomed images were used to observe better this process. The localization and intensity of p62/SQSTM1 staining was also analyzed. DAPI (blue) staining was used for nuclear localization. Images are representative of three independent experiments. Scale bar 30 µm.

Table S4. Analysis of LC3 and p62/SQSTM1 positive area inside A549 cells treated with chloroquine (CQ, 50 µM) and **LAI-1** (15 µM) for 4 and 24 hours. Positive threshold was adjusted to control group (CT) and fold induction against CT group was measured. Results show mean value ± SEM. Statistical differences against CT are showed as *p < 0.05, **p < 0.01, ***p < 0.001 and ****p < 0.0001.

Fold induction of area (Mean ± SEM)	CT	CQ 4h	CQ 24h	LAI-1 4h	LAI-1 24h
**LC3**	1.00 ± 0.00	3.60 ± 0.51*	5.22 ± 0.97***	3.10 ± 0.32	4.89 ± 1.32**
**p62/SQSTM1**	1.00 ± 0.00	1.04 ± 0.13	1.01 ± 0.10	2.63 ± 0.77*	4.23 ± 0.73****

4.3. Necrosis induction by LAI-1 on DMS53 cells

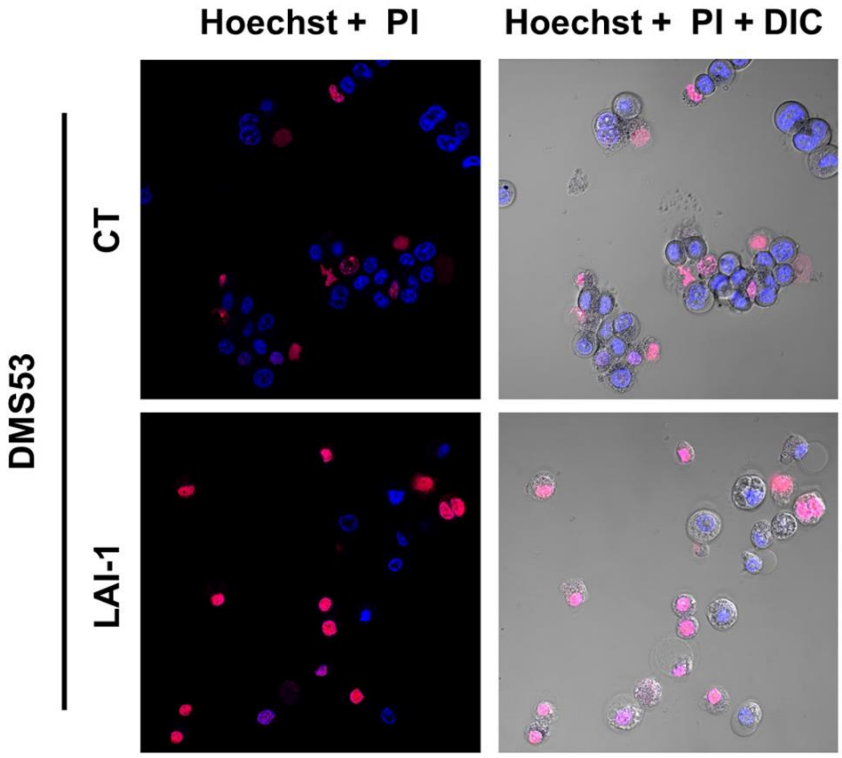

Figure S23. Hoechst and propidium iodide (PI) staining on DMS53 cell line. After 24 h of treatment with **LAI-1** (20 µM) nucleus of cells were stained with Hoechst (Blue) showing viable or apoptotic bodies, and PI (red) staining nucleus without cell membrane integrity. Merged images with differential interference contrast (DIC) were used to visualize cellular structure and cellular membrane shape. Images are representative of three independent experiments. Scale bar 40 µm.

4.4. Original blots from Western blot images

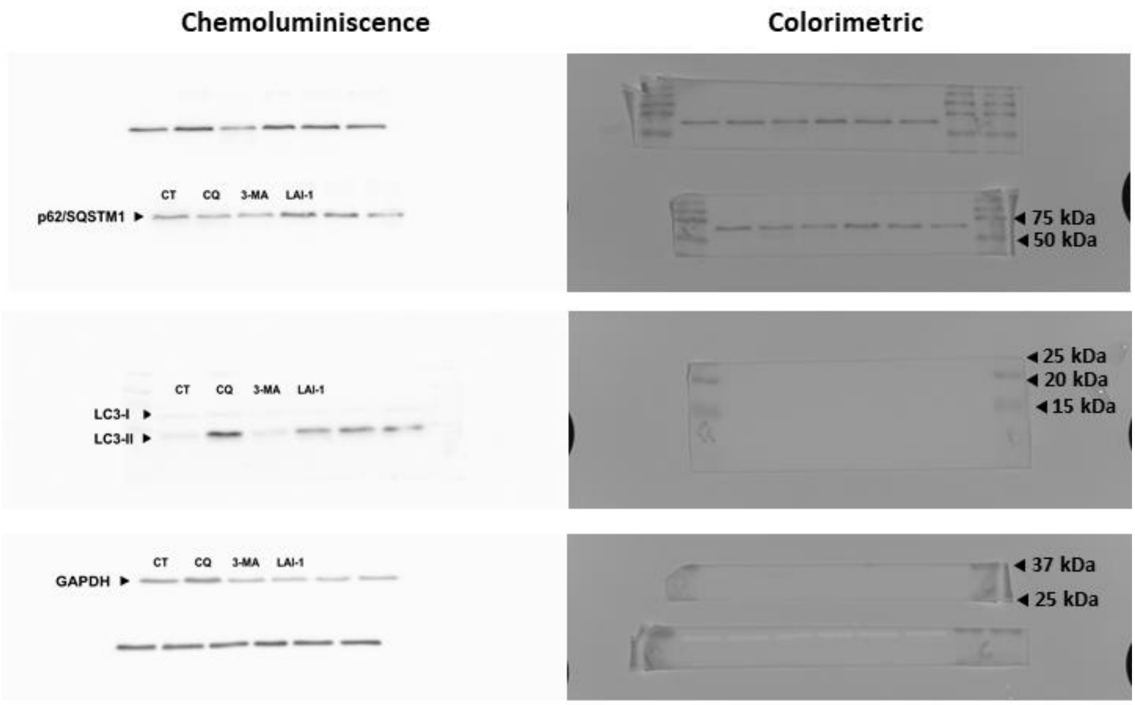

Figure S24. Original blots observed from figure 4A showing p62/SQSTM1, LC3 and GAPDH protein expression on A549 cells. On the left are showed blots after chemiluminescence reaction, whereas on the right are showed the blots after colorimetric capture.

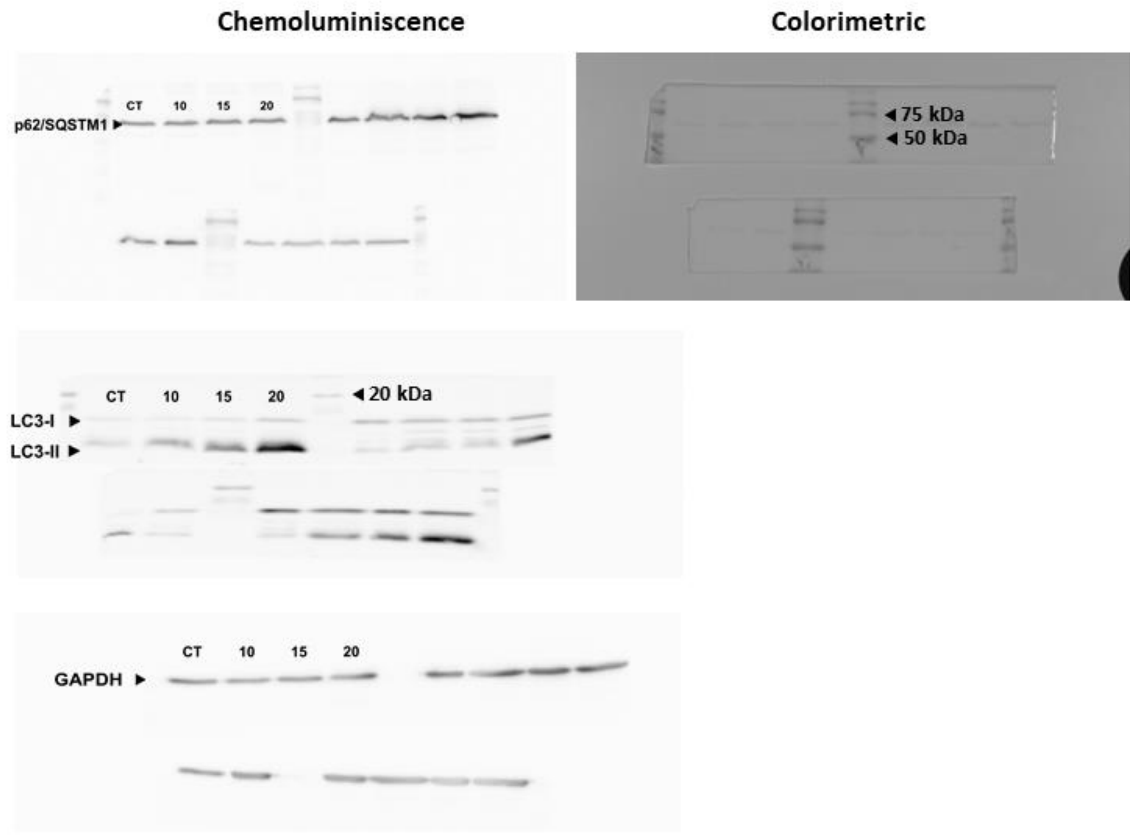

Figure S25. Original blots observed from figure 5A showing p62/SQSTM1, LC3 and GAPDH protein expression on A549 cells. On the left are showed blots after chemiluminescence reaction, whereas on the right are showed the blots after colorimetric capture. Colorimetric images were not captured from LC3 and GAPDH plots.

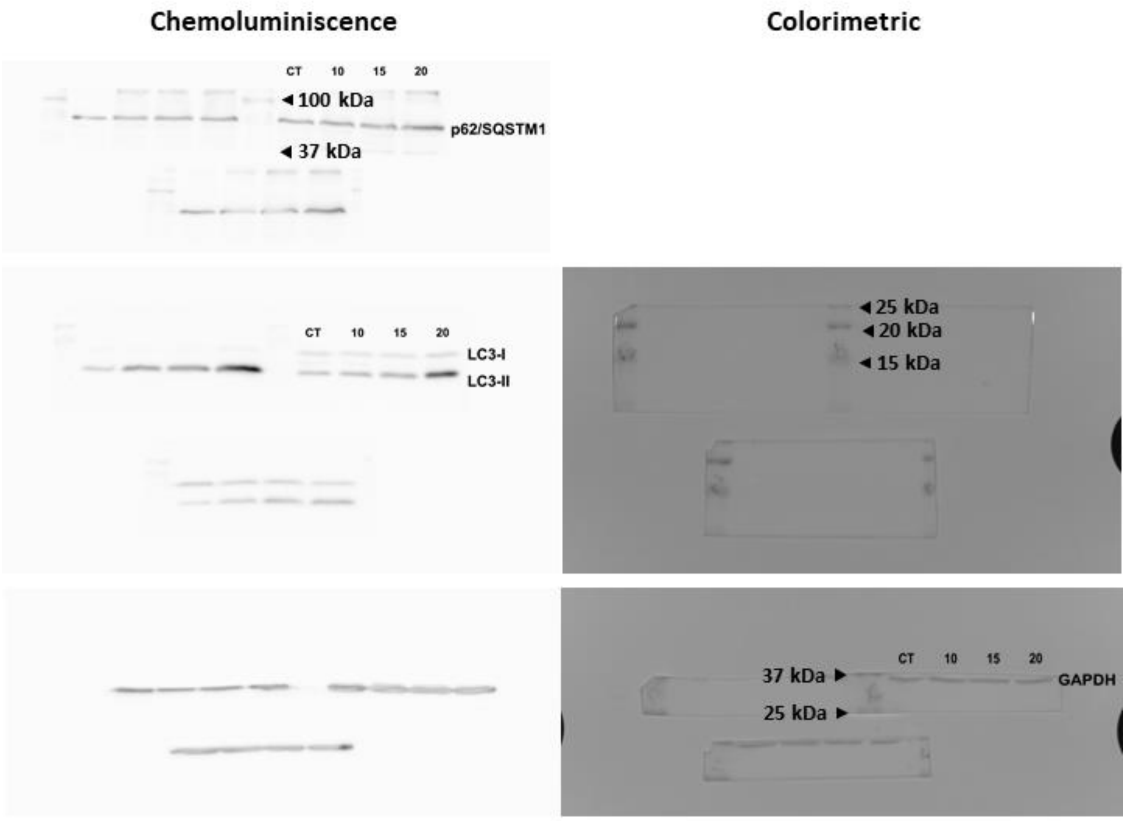

Figure S26. Original blots observed from figure 5A showing p62/SQSTM1, LC3 and GAPDH protein expression on SW900 cells. On the left are showed blots after chemiluminescence reaction, whereas on the right are showed the blots after colorimetric capture. Colorimetric image was not captured from p62/SQSTM1 plot.

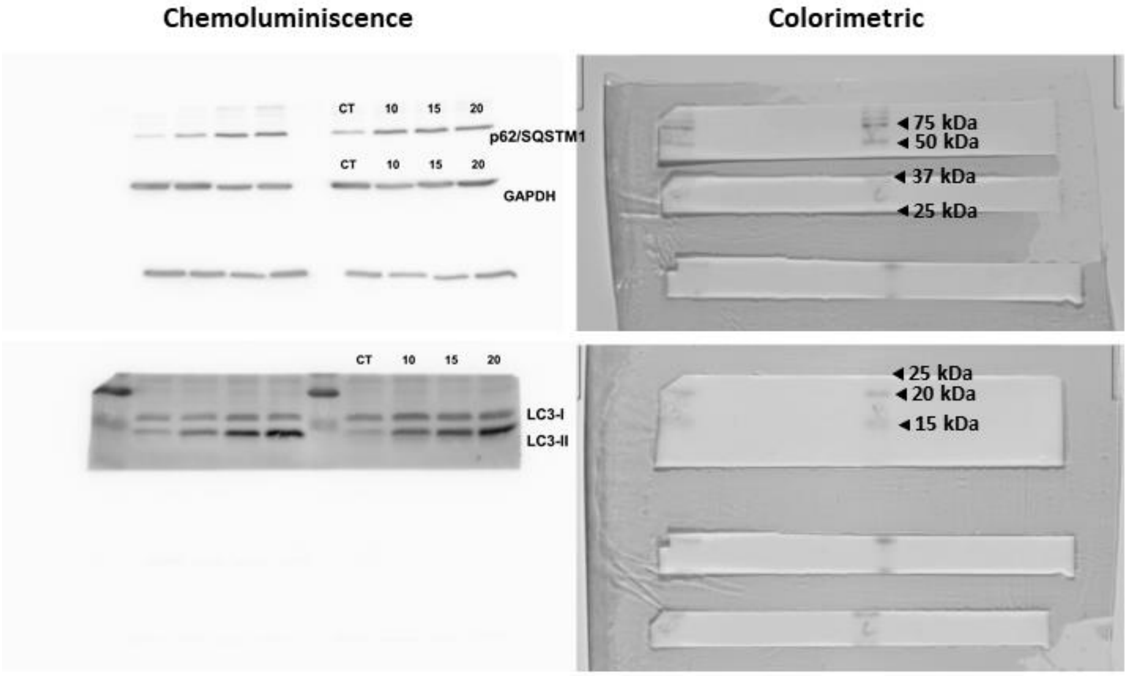

Figure S27. Original blots observed from figure 5A showing p62/SQSTM1, LC3 and GAPDH protein expression on DMS53 cells. On the left are showed blots after chemiluminescence reaction, whereas on the right are showed the blots after colorimetric capture.

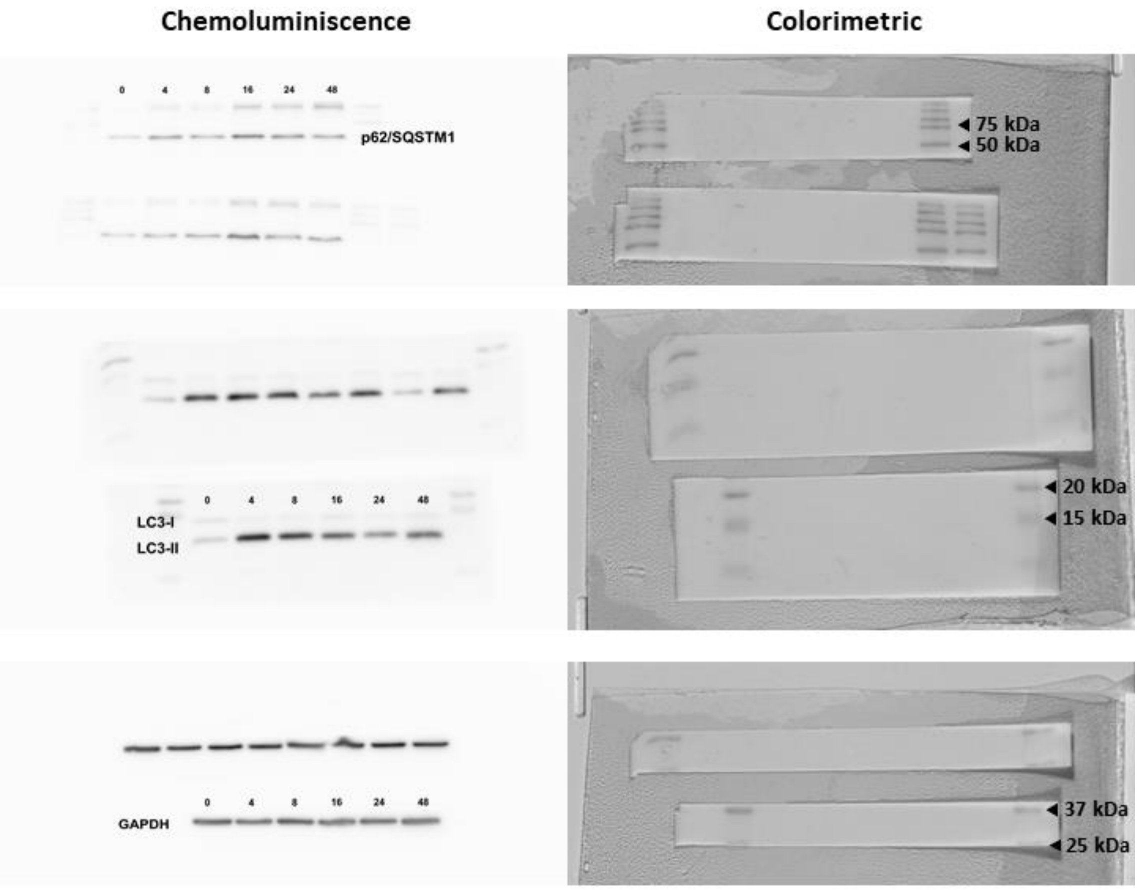

Figure S28. Original blots observed from figure 5D showing p62/SQSTM1, LC3 and GAPDH protein expression on A549 cells. On the left are showed blots after chemiluminescence reaction, whereas on the right are showed the blots after colorimetric capture. Colorimetric image was not captured from p62/SQSTM1 plot.

Chemoluminiscence

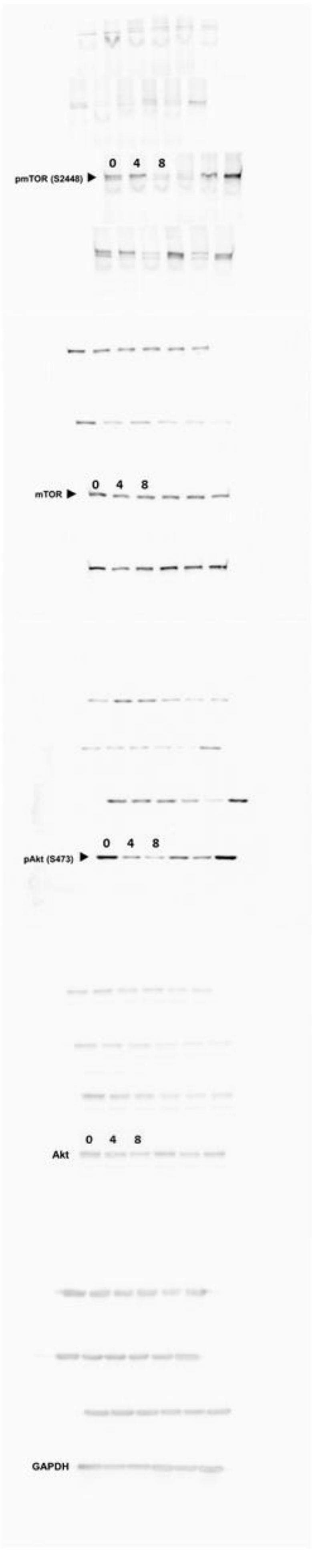

Colorimetric

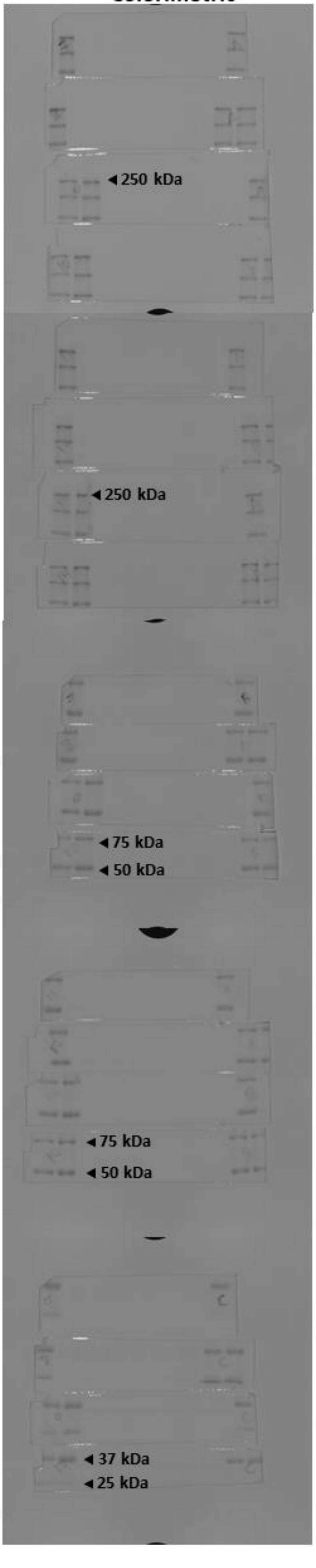

Figure S29. Original blots observed from figure 5G showing p-mTOR (S24888), mTOR, p-Akt (S473), Akt and GAPDH protein expression on A549 cells. On the left are showed blots after chemiluminescence reaction, whereas on the right are showed the blots after colorimetric capture.

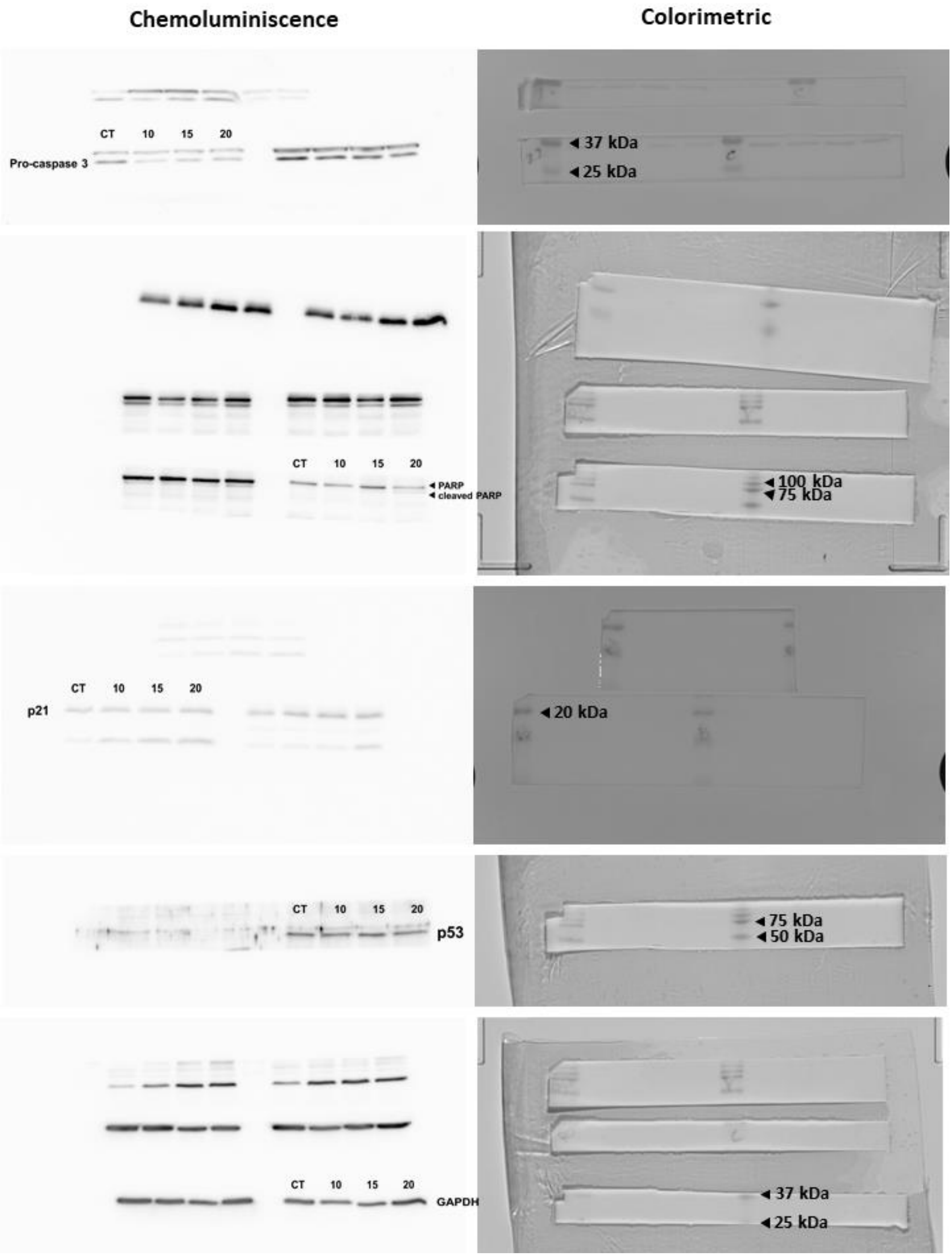

Figure S30. Original blots observed from figure 9A showing pro-caspase 3, PARP, cleaved-PARP, p21, p53 and GAPDH protein expression on A549 cells. On the left are showed blots after chemiluminescence reaction, whereas on the right are showed the blots after colorimetric capture.

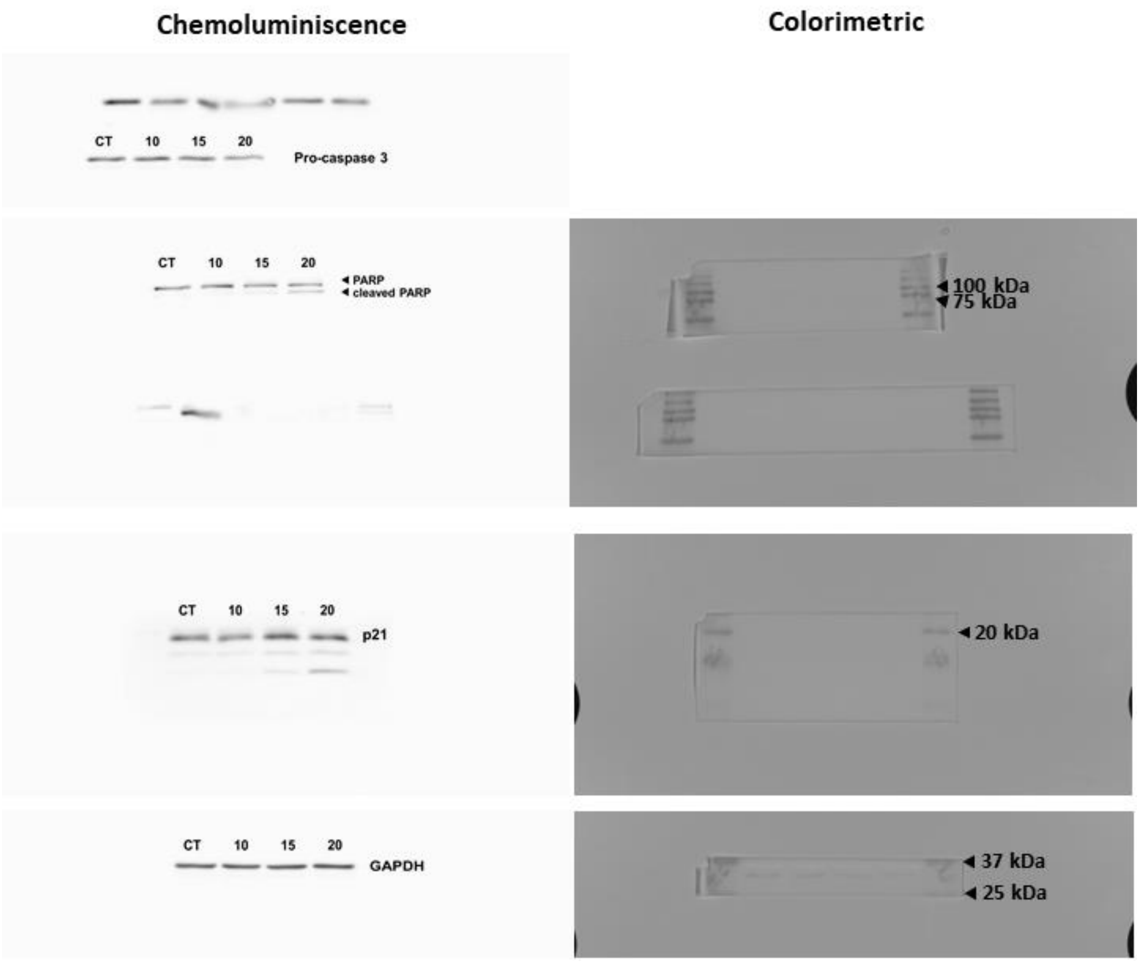

Figure S31. Original blots observed from figure 9A showing pro-caspase 3, PARP, cleaved-PARP, p21, p53 and GAPDH protein expression on SW900 cells. On the left are showed blots after chemiluminescence reaction, whereas on the right are showed the blots after colorimetric capture. Colorimetric image was not captured from pro-caspase 3 plot.

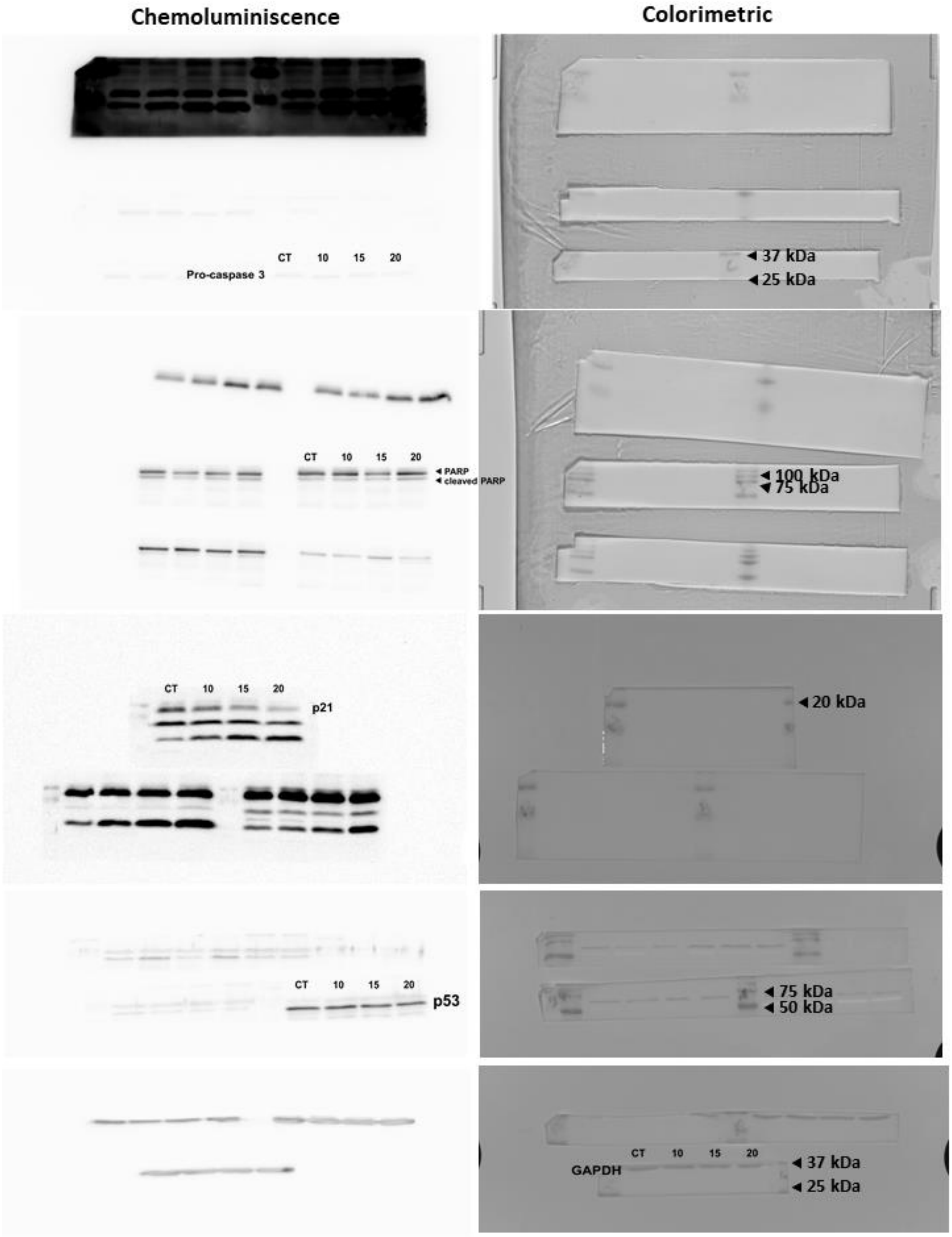

Figure S32. Original blots observed from figure 9A showing pro-caspase 3, PARP, cleaved-PARP, p21, p53 and GAPDH protein expression on DMS53 cells. On the left are showed blots after chemiluminescence reaction, whereas on the right are showed the blots after colorimetric capture.
